# Red-Light-Driven Atom Transfer Radical Polymerization for High-Throughput Polymer Synthesis in Open Air

**DOI:** 10.1021/jacs.3c09181

**Published:** 2023-10-25

**Authors:** Xiaolei Hu, Grzegorz Szczepaniak, Anna Lewandowska-Andralojc, Jaepil Jeong, Bingda Li, Hironobu Murata, Rongguan Yin, Arman Moini Jazani, Subha R. Das, Krzysztof Matyjaszewski

**Affiliations:** † Department of Chemistry, Carnegie Mellon University, Pittsburgh, Pennsylvania 15213, United States; ‡ Faculty of Chemistry, University of Warsaw, Pasteura 1, 02-093 Warsaw, Poland; § Faculty of Chemistry, Adam Mickiewicz University, Uniwersytetu Poznanskiego 8, 61-614 Poznan, Poland; ∥ Center for Advanced Technology, Adam Mickiewicz University, Uniwersytetu Poznanskiego 10, 61-614 Poznan, Poland; ⊥ Center for Nucleic Acids Science & Technology, Carnegie Mellon University, Pittsburgh, Pennsylvania 15213, United States; # Department of Biomedical Engineering, Carnegie Mellon University, Pittsburgh, Pennsylvania 15213, United States

## Abstract

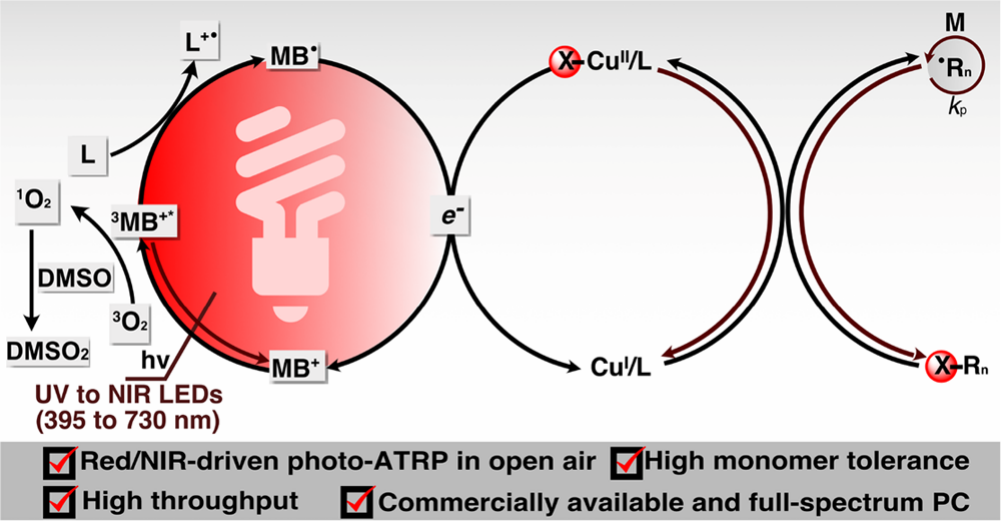

Photoinduced reversible-deactivation radical polymerization (photo-RDRP) techniques offer exceptional control over polymerization, providing access to well-defined polymers and hybrid materials with complex architectures. However, most photo-RDRP methods rely on UV/visible light or photoredox catalysts (PCs), which require complex multistep synthesis. Herein, we present the first example of fully oxygen-tolerant red/NIR-light-mediated photoinduced atom transfer radical polymerization (photo-ATRP) in a high-throughput manner under biologically relevant conditions. The method uses commercially available methylene blue (MB^+^) as the PC and [X–Cu^II^/TPMA]^+^ (TPMA = tris(2-pyridylmethyl)amine) complex as the deactivator. The mechanistic study revealed that MB^+^ undergoes a reductive quenching cycle in the presence of the TPMA ligand used in excess. The formed semireduced MB (MB^•^) sustains polymerization by regenerating the [Cu^I^/TPMA]^+^ activator and together with [X–Cu^II^/TPMA]^+^ provides control over the polymerization. This dual catalytic system exhibited excellent oxygen tolerance, enabling polymerizations with high monomer conversions (>90%) in less than 60 min at low volumes (50–250 μL) and high-throughput synthesis of a library of well-defined polymers and DNA–polymer bioconjugates with narrow molecular weight distributions (*Đ* < 1.30) in an open-air 96-well plate. In addition, the broad absorption spectrum of MB^+^ allowed ATRP to be triggered under UV to NIR irradiation (395–730 nm). This opens avenues for the integration of orthogonal photoinduced reactions. Finally, the MB^+^/Cu catalysis showed good biocompatibility during polymerization in the presence of cells, which expands the potential applications of this method.

## Introduction

The use of light has become a powerful tool for controlling chemical reactions, due to its versatile and noninvasive nature.^1,2^ Reversible-deactivation radical polymerization (RDRP)^3,4^ provides access to well-defined polymers and hybrid materials with complex architectures.^5−7^ Among the various RDRP techniques, photoinduced atom transfer radical polymerization (photo-ATRP)^8^ and photoinduced electron/energy transfer–reversible addition–fragmentation chain transfer (PET-RAFT)^9^ polymerization represent significant advances in polymer science, as they allow precise spatiotemporal and macromolecular chain length control under environmentally benign reaction conditions.^10−15^

ATRP is a reversible redox process in which a halogen is transferred from a dormant C(sp^3^)–X polymer chain end to a [Cu^I^/L]^+^ activator, forming a propagating radical and an [X–Cu^II^/L]^+^ deactivator.^16−18^ Oxygen interferes with the initiation and propagation of radicals by forming peroxy radicals, which is typical of any radical polymerization.^19^ Additionally, O_2_ can oxidize the [Cu^I^/L]^+^ activator in ATRP, quenching the polymerization.^20^ The oxygen sensitivity of conventional ATRP necessitates a laborious degassing process prior to polymerization.^21^ To address these challenges, several oxygen-tolerant ATRP techniques have been developed that rely on activator regeneration.^21−35^ By continuously converting [X–Cu^II^/L]^+^ back to [Cu^I^/L]^+^, the catalytic system acts as an oxygen scavenger, providing oxygen tolerance.^36^ Regeneration of the [Cu^I^/L]^+^ activator can be achieved by reducing agents,^37−41^ enzymes,^42^ electrostimuli,^43−45^ photostimuli,^46^ or mechanochemical stimuli.^47,48^

Initially, Cu-catalyzed photo-ATRP was performed using UV light irradiation.^8,49,50^ In this process, the excited Cu(II) complex is reduced by an electron donor, such as an amine-based ligand used in excess.^46^ The formed [Cu^I^/L]^+^ activator then reacts with a C(sp^3^)–X polymer chain end to yield a carbon-based radical and [X–Cu^II^/L]^+^ deactivator. However, UV light has a biocidal effect on biomacromolecules and can cause undesirable side reactions.^51^ Alternatively, the dormant polymer chain can be activated by direct electron transfer from an organophotoredox catalyst under visible light irradiation,^52−55^ but organocatalyzed ATRP (O-ATRP) is mainly limited to methacrylates and organic solvents.^56−59^ To overcome these limitations, photoredox/copper catalytic systems have been developed,^60^ although most of them operate only in the wavelength range of 400 to 520 nm.^61−70^

The use of low-energy red/near-infrared (NIR) light has become increasingly popular in polymer chemistry due to its high biocompatibility, penetrability, reduced scattering, and minimal side reactions.^71,72^ Its potential applications include 3D printing,^73^ polymerization-induced self-assembly (PISA),^74^ and polymerization in vivo and through barriers.^75−77^ Therefore, the development of photo-RDRP systems with suitable photoredox catalysts (PCs) that can trigger RDRP under long-wavelength light is desirable. There are several examples of red/NIR-light-mediated RDRP using PCs, such as bacteriochlorophyll,^78^ porphyrines,^79^ phthalocyanines,^74,80−82^ conjugated porphyrin^83^ for RAFT polymerization and Zn porphyrin,^84^ conjugated phenothiazines or phosphine,^63,85^ cyanines,^86,87^ or upconverting nanoparticles for ATRP.^77,88^ However, most of these photocatalysts are not commercially available, require multistep synthesis, or are only soluble in organic solvents. In addition, the long reaction time and limited oxygen tolerance hinder the application of these systems.

High-throughput screening in chemical synthesis improves efficiency and productivity and reduces costs.^89,90^ In recent years, significant progress has been made in combining RDRP techniques with high-throughput methods, allowing rapid optimization of reaction parameters and synthesis of a diverse library of polymers with different properties.^91^ A key requirement for this combinatorial approach is improved oxygen tolerance during polymerization.^21,22^ Recently, high-throughput synthesis of well-defined high molecular weight polymers via enzymatically degassed RAFT polymerization in a 96-well plate was reported.^92^ There are also several examples of performing PET-RAFT in a high-throughput manner,^93−96^ but similar ATRP systems are still rare.^97^

**Figure 1:**
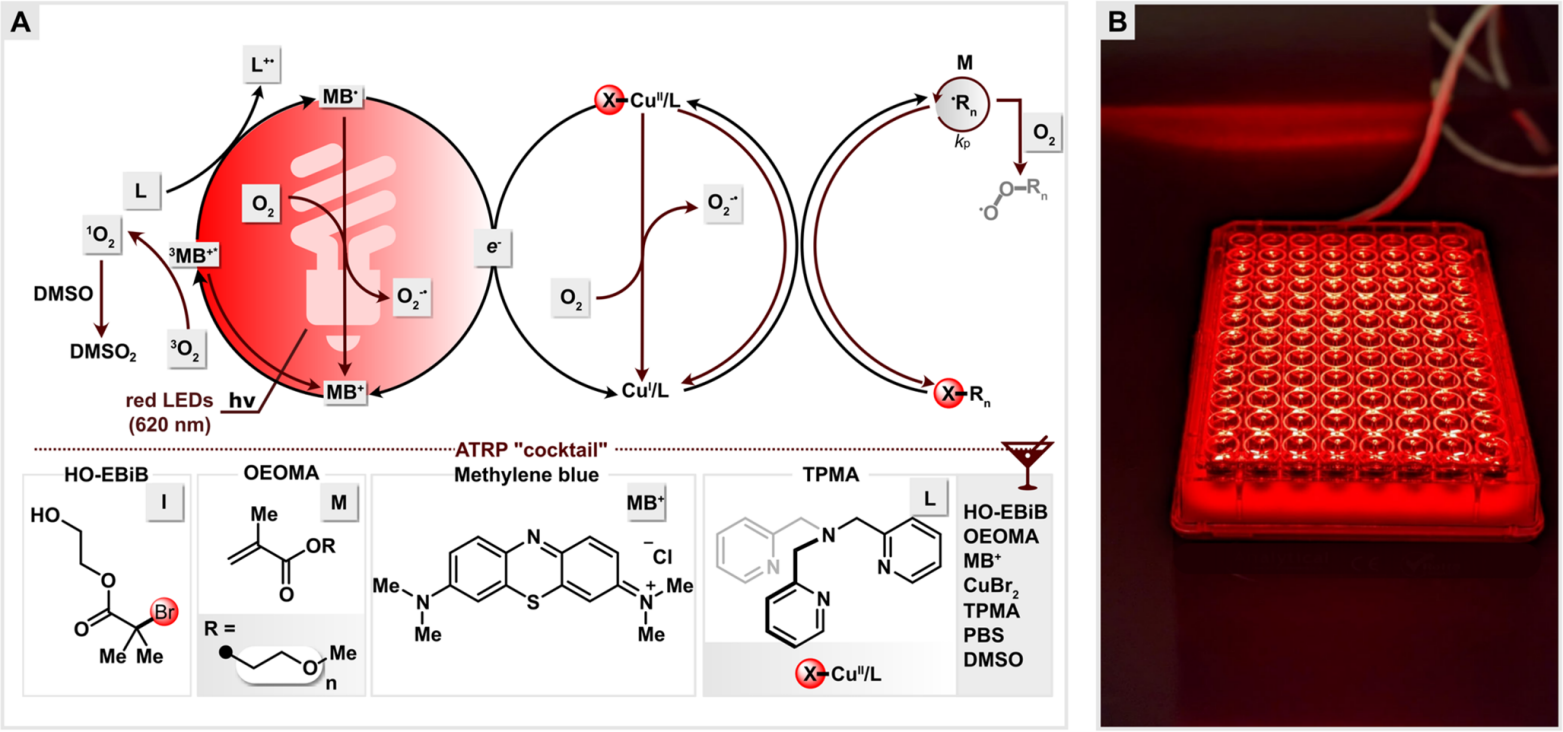
(A) Reductive quenching mechanism of MB^+^/Cu-catalyzed photo-ATRP; (B) high-throughput reaction setup in a 96-well plate.

Herein, we report red-light-mediated ATRP using commercially available methylene blue (MB^+^) as the PC and [X–Cu^II^/TPMA]^+^ (TPMA = tris(2-pyridylmethyl)amine) complex as the deactivator (Figure 1A). MB^+^ is a water-soluble and biocompatible cationic dye (note that in the literature MB^+^ is often abbreviated as MB, without a positive charge) that is commonly used as a PC in organic and polymer synthesis.^98−103^ Despite these attractive properties, it has not yet been successfully used in photo-ATRP due to its high oxidation potential.^104^ The developed dual-catalytic ATRP system exhibited excellent oxygen tolerance, allowing open-air polymerizations with high monomer conversions (>90%) in less than 60 min at low volumes (50–250 μL). The method was applied to the high-throughput synthesis of a library of well-defined polymers and DNA–polymer bioconjugates with low dispersities (*Đ* < 1.30) in an open-air 96-well plate in both water and dimethyl sulfoxide (DMSO). Finally, the MB^+^/Cu catalysis showed good biocompatibility during polymerization in the presence of cells, expanding the potential biological applications of this method.

## Results and Discussion

### Polymerization Conditions

Oligo(ethylene oxide) methyl ether methacrylate (OEOMA_500_, average *M*_n_ = 500) was polymerized in a phosphate-buffered saline (PBS) solution containing DMSO (10% v/v) (Figure 1A). PBS was chosen as the reaction medium to ensure benign conditions and to prevent loss of control over the ATRP due to the dissociation of the [X–Cu^II^/L]^+^ deactivator to the [Cu^II/^L]^2+^ complex and a free halide anion.^105,106^ The photo-ATRP was performed in an open-air 96-well plate using a 96-point LED array (630 nm, 25 mW cm^–2^, Figure 1B). 2-Hydroxyethyl α-bromoisobutyrate (HO-EBiB) was used as the initiator, MB^+^ as the organophotoredox catalyst, [X–Cu^II^/TPMA]^+^ complex as the deactivator, and an excess of TPMA as the electron donor (ED) (Figure 1A, the mechanism of the MB^+^/Cu dual catalysis is discussed later).

Several key experiments were performed to investigate the effect of the ATRP “cocktail” components on polymerization performance (Table 1, entries 1–5). The exclusion of the HO-EBiB initiator led to an uncontrolled polymerization (Table 1, entry 1). In the absence of MB^+^, irradiation with red light resulted in no polymerization as measured by ^1^H NMR (Table 1, entry 2). The same result was obtained when MB^+^ was used without the copper catalyst (Table 1, entry 3). Direct activation of the dormant polymer chain end (C(sp^3^)–X) by excited MB^+^ was not possible, due to its high oxidation potential.^104,107^ Furthermore, no monomer conversion was observed in the absence of the TPMA ligand. However, when CuBr_2_, TPMA, and MB^+^ were used in a molar ratio of [OEOMA_500_]/[HO-EBiB]/[MB^+^]/[CuBr_2_]/[TPMA] = 200/1/0.05/0.2/0.6, a well-controlled polymerization (*Đ* = 1.23) with a molecular weight of *M*_n,th_ = 75 000 and *M*_n,MALS_ = 62 600 was achieved, reaching 75% monomer conversion within 30 min (Table 1, entry 5). These results highlight the critical role of the MB^+^/Cu dual-catalytic system. Further optimization was performed by adjusting the MB^+^ concentration. Using MB^+^ at a concentration of 7.5 μM (25 ppm relative to that of OEOMA_500_), the polymerization proceeded slowly with only 7% monomer conversion achieved after 30 min (Table 1, entry 6). Increasing the MB^+^ concentration by 2- and 5-fold (50 and 125 ppm with respect to the monomer) significantly increased the monomer conversion to 32% and 72%, respectively (Table 1, entries 7 and 8). The effect of the copper concentration on the polymerization performance was then investigated. As expected, higher concentrations of the [X–Cu^II^/L]^+^ deactivator allowed better control over polymerization, with dispersity values decreasing from 1.29 to 1.15 when the copper concentration was increased from 0.075 to 0.45 mM (Table 1, entries 8–11). Finally, the concentration of the TPMA ligand, which also acted as an electron donor in the MB^+^/Cu catalytic system, was optimized (Table 1, entry 12). When TPMA was increased from 0.9 to 1.2 mM, the monomer conversion increased and the control over the molecular weight (MW) improved (*M*_n,th_ = 71 000, *M*_n,MALS_ = 70 500).

**Table 1:** Optimization of Polymerization Conditions^a^

entry	HO-EBiB (equiv)	MB^+^ (equiv)	CuBr_2_ (equiv)	TPMA (equiv)	conv.^b^ (%)	*M*_n,th_	*M*_n,app_^c^	*M*_n,MALS_^d^	*Đ*^c^
1		0.05	0.2	0.6	18		129 200		1.47
2	1		0.2	0.6	0				
3	1	0.05		0.6	0				
4	1	0.05	0.2		0				
5	1	0.05	0.2	0.6	75	75 000	44 400	62 600	1.23
6	1	0.005	0.2	0.6	7	7000	13 700	9000	1.23
7	1	0.01	0.2	0.6	32	32 000	26 700	29 700	1.22
8	1	0.025	0.2	0.6	72	72 000	41 800	55 500	1.19
9	1	0.025	0.05	0.6	89	89 000	57 800	90 300	1.29
10	1	0.025	0.1	0.6	84	84 000	52 600	75 900	1.24
11	1	0.025	0.3	0.6	69	69 000	44 000	59 700	1.15
12	1	0.025	0.3	0.9	71	71 000	51 900	70 500	1.17

a Reaction conditions: [OEOMA_500_]/[HO-EBiB]/[MB^+^]/[CuBr_2_]/[TPMA] = 200/1/*x*/*x*/*x*, [OEOMA_500_] = 300 mM, in 1× PBS with DMSO (10% v/v), irradiated for 30 min under red LEDs (630 nm, 25 mW cm^–2^) in an open-air 96-well plate at a volume of 250 μL.

b Monomer conversion was determined using ^1^H NMR spectroscopy.

c Molecular weight (*M*_n,app_) and dispersity (*Đ*) were determined by size exclusion chromatography (SEC) analysis (DMF as eluent) calibrated to poly(methyl methacrylate) standards.

d Absolute molecular weight (*M*_n,MALS_) was determined by SEC analysis (DMF as eluent) with a multiangle light scattering (MALS) detector.

### Kinetic Study

**Figure 2:**
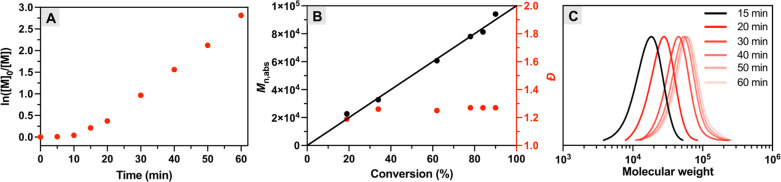
MB^+^/Cu-catalyzed photo-ATRP in a 96-well plate: (A) first-order kinetic plot, (B) evolution of molecular weight and molecular weight distribution with monomer conversion, and (C) SEC traces evolution with time. Reaction conditions: [OEOMA_500_]/[HO-EBiB]/[MB^+^]/[CuBr_2_]/[TPMA] = 200/1/0.025/0.3/0.9, [OEOMA_500_] = 300 mM, in 1× PBS with DMSO (10% v/v), irradiated for 60 min under red LEDs (630 nm, 25 mW cm^–2^) in an open-air 96-well plate at a volume of 250 μL.

The MB^+^/Cu-catalyzed photo-ATRP was performed under optimized conditions (Table 1, entry 12) in an open-air 96-well plate in a high-throughput manner (Figure S2). A stock solution of the ATRP “cocktail” was prepared and transferred to each well. Polymerization samples were quenched with 1,4-bis(3-isocyanopropyl)piperazine at the desired time and then taken from each well for ^1^H NMR measurement (Figure S3).^108^ Kinetic analysis showed a short induction period (∼10 min), corresponding to the time required for the catalytic system to remove oxygen from the polymerization mixture, followed by rapid polymerization, reaching 90% monomer conversion within 60 min (Figure 2A). The molecular weight increased as a function of monomer conversion in excellent agreement with theoretical values, and the dispersity values remained low (*Đ* < 1.27) (Figure 2B). In addition, the monomodal SEC traces shifted toward the high-MW region as polymerization progressed (Figure 2C). Similar polymerization kinetics were observed at varying MB^+^ concentrations (Figure S4).

### Varying Targeted Degrees of Polymerization

**Figure 3:**
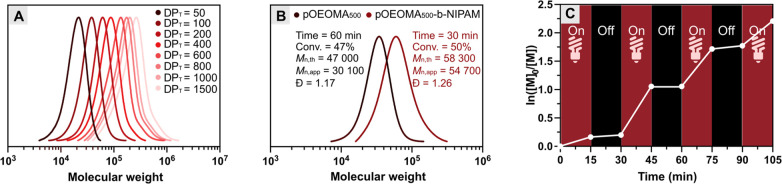
(A) SEC traces of pOEOMA_500_ with different targeted degrees of polymerization, (B) chain extension of pOEOMA_500_ macroinitiator with NIPAM, and (C) temporal control of MB^+^/Cu-catalyzed photo-ATRP.

Achieving well-defined polymers over a wide MW range using RDRP techniques in an open-air 96-well plate remains a significant challenge. To address this issue, MB^+^/Cu-catalyzed photo-ATRP was further investigated for modulating the molecular weights (Table 2 and Figure 3A). By adjusting the initiator concentration, various degrees of polymerization (DP_T_) up to 1500 were targeted while keeping the other polymerization components at a constant concentration. As shown in Table 2, the monomer conversion reached 50–70% within 30 min in all cases. In addition, the molecular weights (*M*_n,MALS_) of the resulting polymers showed good agreement with theoretical values with relatively low dispersity (*Đ* < 1.40). This demonstrates the high degree of control over the synthesis of polymers with varying MWs. Alternatively, adjusting the monomer concentration while keeping the concentrations of the other components constant also allowed the synthesis of polymers with low dispersity values (*Đ* < 1.20) and controlled MWs in all cases (Table S1).

**Table 2:** Polymerization of OEOMA_500_ with Varying Degrees of Polymerization^a^

entry	DP_T_	[HO-EBiB] (mM)	conv. (%)^b^	*M*_n,th_	*M*_n,app_^c^	*M*_n,MALS_^d^	*Đ*^c^
1	50	6.0	61	15 250	18 500	20 400	1.19
2	100	3.0	71	35 500	31 600	32 500	1.20
3	200	1.5	71	71 000	51 900	70 500	1.17
4	400	0.75	63	126 000	76 600	135 000	1.21
5	600	0.5	62	186 000	110 700	181 300	1.26
6	800	0.375	59	236 000	132 600	222 800	1.29
7	1000	0.3	58	290 000	156 700	233 100	1.29
8	1500	0.2	51	382 500	194 200	321 200	1.39

a Reaction conditions: [OEOMA_500_] = 300 mM, [HO-EBiB] = 0.2–6.0 mM, [MB^+^] = 37.5 μM, [CuBr_2_] = 0.45 mM, [TPMA] = 1.35 mM, in 1× PBS with DMSO (10% v/v), irradiated for 30 min under red LEDs (630 nm, 25 mW cm^–2^) in an open-air 96-well plate at a volume of 250 μL.

b Monomer conversion was determined by using ^1^H NMR spectroscopy.

c Molecular weight (*M*_n,app_) and dispersity (*Đ*) were determined by SEC analysis (DMF as eluent) calibrated to poly(methyl methacrylate) standards.

d Absolute molecular weight (*M*_n,MALS_) was determined by SEC analysis (DMF as eluent) with a multiangle light scattering (MALS) detector.

### Chain Extension

To confirm the chain-end fidelity of the polymers synthesized by MB^+^/Cu-catalyzed photo-ATRP, pOEOMA_500_ (*M*_n,app_ = 30 100, *Đ* = 1.17) was first synthesized and then used as a macroinitiator for chain extension with *N*-isopropylacrylamide (NIPAM). The resulting block copolymer had a molecular weight (*M*_n,app_) of 54 700 and a low dispersity of 1.26. In addition, the SEC traces were clearly shifted to the higher MW region without tailing and without a shoulder peak in the lower MW region (Figure 3B). A similar phenomenon (Figure S5) was observed when pOEOMA_500_ was chain extended with OEOMA_500_, yielding pOEOMA_500_-*b*-pOEOMA_500_ (*M*_n,MALS_ = 92 800, *Đ* = 1.27), which was in agreement with the theoretical value (*M*_n,th_ = 97 800).

### Temporal Control

Temporal control of the polymerization was achieved by switching the red light on and off, as shown in Figure 3C. When the light was turned off, negligible or no monomer conversion was observed, because oxygen from the air constantly diffusing into the aqueous polymerization mixture oxidized the [Cu^I^/L]^+^ activator to its inactive form (Table S2). When the light was turned on, the MB^+^/Cu catalysis rapidly removed the oxygen and regenerated the activator, restarting the polymerization. Multiple on/off cycles of red light did not hamper the temporal control.

### Mechanistic Study

To gain deeper insight into the photo-ATRP mechanism using MB^+^ as an organophotoredox catalyst together with the [Br–Cu^II/^TPMA]^+^ complex, time-resolved emission experiments and nanosecond flash photolysis were performed to determine the reactivity and kinetics of the primary photochemical steps upon MB^+^ excitation.

**Figure 4:**
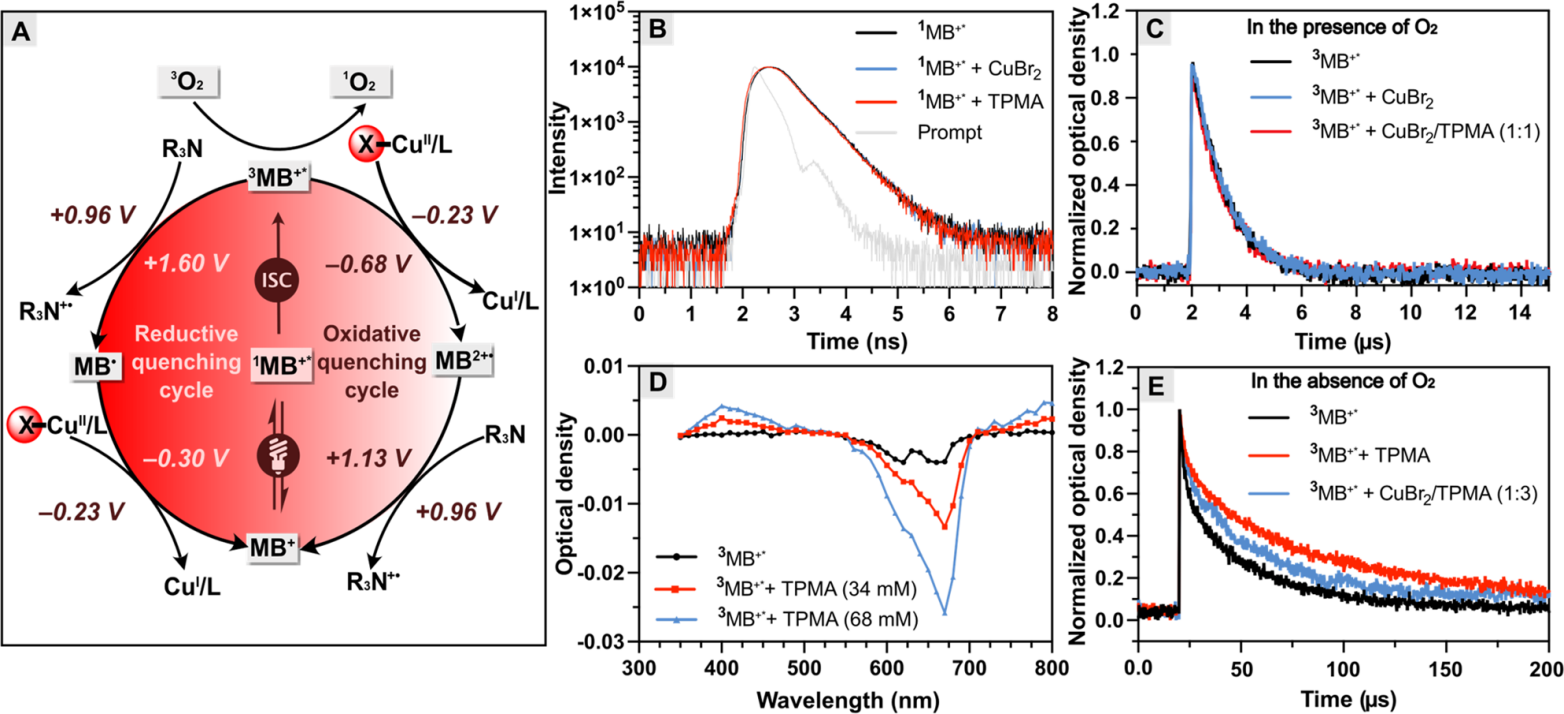
(A) Reductive quenching vs oxidative quenching cycle for MB^+^/Cu-catalyzed photo-ATRP, (B) decay of MB^+^ (37.5 μM) fluorescence recorded in the absence and in the presence of CuBr_2_ (34 mM) or TPMA (34 mM), and prompt (gray, instrument response function), λ_ex_ = 640 nm, λ_em_ = 672 nm, (C) transient absorption decay profiles at 420 nm for the air-saturated MB^+^ solution (37.5 μM) and in the presence of CuBr_2_ (34 mM) or CuBr_2_/TPMA (1:1 molar ratio, 34 mM) after the excitation at 532 nm, (D) comparison of the transient UV–vis absorption spectra obtained during laser flash photolysis with 200 μs time delay (with excitation at 532 nm) of deoxygenated MB^+^ solution (37.5 μM) and in the presence of TPMA (34 or 68 mM), and (E) transient absorption decay profiles at 420 nm for the deoxygenated MB^+^ and in the presence of TPMA (34 mM) or CuBr_2_/TPMA (1:3 molar ratio, [TPMA] = 34 mM) after the excitation at 532 nm.

The visible light irradiation excites the ground state MB^+^ to the singlet excited state MB^+^ (^1^MB^+^*), which undergoes efficient intersystem crossing (ISC) to generate the excited triplet state MB^+^ (^3^MB^+^*), which can act as either a reducing or an oxidizing agent (Figure 4A).

First, the possible interaction of ^1^MB^+^* with TPMA or CuBr_2_ was investigated. The fluorescence lifetime of MB^+^ was obtained from the monoexponential fit of its fluorescence decay signal (Figure 4B). It was approximately 350 ps, which is in good agreement with the previously reported fluorescence lifetime of MB^+^ in water.^109^ With the addition of TPMA (34 mM) or CuBr_2_ (11 mM), the decay kinetics of MB^+^ fluorescence remained unchanged, indicating that TPMA or CuBr_2_ did not quench the singlet excited state of methylene blue (Figure 4B).

Upon laser excitation at 532 nm, the methylene blue solution exhibited a broad and strong bleaching band that was identical to the ground-state band at 660 nm (Figure S6). In addition, a band of positive signals with a maximum at 410 nm and a second band in the 700–800 nm range were observed in the transient spectra, which were attributed to the formation of ^3^MB^+^*. The lifetime of ^3^MB^+^* was determined by a monoexponential fit of the bleach recovery at 640 nm. In an oxygen-free solution, the triplet lifetime was estimated to be 31.2 μs (Figure S7A). In the presence of oxygen, the triplet excited state showed rapid deactivation following first-order kinetics, with a lifetime of 1.76 μs (Figure S7B). The main deactivation process of ^3^MB^+^* in the absence of the [Br–Cu^II/^TPMA]^+^ complex was its reaction with ^3^O_2_, leading to the formation of ^1^O_2_ and MB^+^ in the ground state. Considering the solubility of oxygen in aqueous solution (0.26 mM), the rate constant for the energy transfer process between ^3^MB^+^* and ^3^O_2_ was estimated to be 2.8 × 10^8^ M^–1^ s^–1^. In the absence of TPMA or CuBr_2_, ^3^MB^+^* was the only intermediate observed in air- or argon-saturated solution. In the air-saturated solution, the energy transfer to ^3^O_2_ was the major quenching pathway.

Since ^3^MB^+^* can act either as a reductant or as an oxidant (see the thermodynamic calculations in the SI), it was first investigated whether ^3^MB^+^* can be oxidatively quenched by the CuBr_2_ or [Br–Cu^II^/TPMA]^+^ complex (Figure 4A). After the addition of CuBr_2_ or CuBr_2_/TPMA at a molar ratio of 1:1, the decay kinetics of the ^3^MB^+^* at 420 nm remained unchanged (Figure 4C), indicating that CuBr_2_ and [Br–Cu^II^/TPMA]^+^ complex (CuBr_2_/TPMA = 1:1) do not act as the ^3^MB^+^* quenchers.

Another possible pathway is the reduction of ^3^MB^+^* by an excess of TPMA (R_3_N, an electron donor) through a reductive quenching cycle (Figure 4A). The interaction of the excited state of ^3^MB^+^* with TPMA was then investigated. The transient absorption spectrum of ^3^MB^+^* in the presence of TPMA was virtually identical to that obtained for ^3^MB^+^* alone (Figure S8).^110^ However, flash photolysis of the oxygen-free solution of MB^+^ in the presence of TPMA resulted in a relatively longer-lasting transient absorption with a maximum at 420 nm (Figure 4D). This absorption could not be attributed to ^3^MB^+^* because its triplet state decayed faster under the same experimental conditions (Figure S6). The relatively slow decay of ^3^MB^+^* in the presence of TPMA and the increase in intensity of the absorption maximum at 420 nm with increasing TPMA concentration suggest that the absorption is due to the semireduced form of the MB (MB^•^) (Figure 4D,E).^111^ To probe the oxidation of the MB^•^ by the [Br–Cu^II^/TPMA]^+^ complex, nanosecond flash photolysis was performed in the presence of CuBr_2_ and an excess of TPMA. It was found that the absorption band at 420 nm, attributed to the MB^•^, decayed faster in the presence of the [Br–Cu^II^/TPMA]^+^ complex (Figure 4E).

To further confirm the reductive quenching cycle in the MB^+^/Cu dual-catalytic system (Figure 4A), steady-state irradiation of argon-saturated MB^+^ in the presence of TPMA was performed and monitored by UV–vis measurement (Figure S9). It shows a clear transformation of the ground-state MB^+^ with blue color to the colorless reduced form of methylene blue in the presence of TPMA alone under light irradiation. Interestingly, the UV–vis spectra confirmed that the oxidized form was restored when oxygen was introduced into the solution by opening the valve cap. Alternatively, the reduced form of methylene blue was oxidized upon the addition of CuBr_2_. Therefore, it is concluded that MB^+^ undergoes a reductive quenching cycle in the presence of TPMA by accepting an electron, generating the MB^•^ with an absorption band at 420 nm and the oxidized TPMA ligand (R_3_N^+•^).^110,112−114^

The quenching rate constant of ^3^MB^+^* by TPMA could not be directly determined by nanosecond flash photolysis because of the significant overlap between the transient absorption spectrum of ^3^MB^+^* and MB^•^. To estimate the quenching rate constant, the formation of singlet oxygen in the absence and presence of TPMA was monitored in real time by time-resolved measurements of its phosphorescence. The time-resolved measurements (decay traces at 1270 nm) were obtained using a so-called “burst mode”, in which the sample is first excited by multiple laser pulses to generate singlet oxygen and then allowed to decay in the 100 μs time window. Figures S10 and S11 show the time-resolved ^1^O_2_ decay for MB^+^ alone and MB^+^ in the presence of TPMA, respectively. The addition of TPMA (11 mM) decreased the amplitude of the ^1^O_2_ phosphorescence by 7%. Based on this, the quenching rate constant *k*_q_ of ^3^MB^+^* by TPMA was estimated to be 4.5 × 10^6^ M^–1^ s^–1^. This value is in the similar range to that reported for ^3^MB^+^* quenching by pyrrolidine (*k*_q_ = 7.1 × 10^6^ M^–1^ s^–1^) or diethylamine (*k*_q_ = 7.1 × 10^6^ M^–1^ s^–1^).^108^

In summary, the excess of TPMA ligand acting as a sacrificial electron donor reductively quenches ^3^MB^+^* (*E*_1/2_(^3^MB^+^*/MB^•^) = +1.60 V vs SCE) (Figure 4A). This results in the formation of the semireduced MB radical (MB^•^) and an amine radical cation (R_3_N^+•^). Subsequently, MB^•^ (*E*_1/2_(MB/MB^•^) = −0.30 V vs SCE) reduces Cu^II^/L (*E*_1/2_(Cu^II^/ Cu^I^) = −0.23 V vs SCE) by electron transfer, generating the Cu^I^/L complex and reforming the MB^+^ in the ground state. Control over radical propagation is achieved by a reversible redox equilibrium between Cu^I^/Cu^II^ complexes, where they intermittently activate dormant species and deactivate radicals. The formed R_3_N^+•^ can undergo deprotonation at the α-position to generate a highly reactive intermediate, the α-amino radical.^1,115,116^ This radical can then further undergo single electron transfer to form an iminium ion and concurrently reduce another Cu^II^/L to Cu^I^/L. The radical can also initiate a new polymer chain, terminate a propagating radical, or react with oxygen.^63,117,118^ MB^+^ photocatalyst and an excess of the TPMA ligand used as the ED, together with the [X–Cu^II^/TPMA]^+^ deactivator, are essential for the induction and to sustain ATRP.

### Oxygen Scavenging

There are three possible pathways for scavenging molecular oxygen (^3^O_2_) by the MB^+^/Cu dual-catalytic system to prevent the formation of unreactive chain-end peroxy radicals in open-air photo-ATRP (Figure 1A): (a) energy transfer from ^3^MB^+^* to form ^1^O_2_ (Figure S7), (b) electron transfer from MB^•^ to generate superoxide anion (O_2_^•–^), and (c) electron transfer from [Cu^I^/L]^+^ complex to form O_2_^•–^.^35,97^

To determine the dominant scavenging pathway and products (^1^O_2_ vs O_2_^•–^), the quenching rates of ^3^MB^+^* by ^3^O_2_ and by the TPMA ligand were compared. Oxygen scavenging by MB^•^ and [Cu^I^/L]^+^ relies on prior reduction of ^3^MB^+^* by the sacrificial electron donor (TPMA). Based on the quenching rate constant of ^3^MB^+*^ by ^3^O_2_ and by TPMA (*k*_q_ = 2.8 × 10^8^ M^–1^ s^–1^ and 4.5 × 10^6^ M^–1^ s^–1^, respectively) and the concentration of ^3^O_2_ (0.26 mM) and TPMA (1.35 mM), the quenching of ^3^MB^+*^ by ^3^O_2_ was at least 12 times faster than by TPMA, indicating that ^3^MB^+^* is the major ^3^O_2_ scavenger in the MB^+^/Cu dual-catalytic system.

In addition, the pathway for ^3^O_2_ removal can also be elucidated by quantifying the percentage of new chains generated during polymerization by comparing the measured molecular weights (*M*_n,MALS_) with the theoretical values (*M*_n,th_) (Table 2). The superoxide anion O_2_^•–^ formed by electron transfer from MB^•^ or [Cu^I^/L]^+^ to ^3^O_2_ can be further reduced in water to hydrogen peroxide (H_2_O_2_). H_2_O_2_ can then react with [Cu^I^/L]^+^ via a Fenton-type reaction to form hydroxyl radical, which can subsequently generate a new chain.^27^ In turn, ^1^O_2_ formed by energy transfer from ^3^MB^+^* in the presence of DMSO (under typical conditions [DMSO]/[O_2_] = 5400) can form inert dimethyl sulfone (DMSO_2_).^119,120^

The molecular weight analysis showed that less than 8% of new chains were generated during the synthesis of polymers with DP_T_ values up to 800 (Table 2), which correlates well with our previous results. For example, less than 1% of new chains were formed at DP_T_ = 200. This is consistent with the formation of “innocent” ^1^O_2_, which does not lead to the formation of new chains. However, for higher DP_T_ values (1000 and 1500), the percentage of newly generated chains increased, suggesting that O_2_^•–^ formation became more significant at low concentrations of HO-EBiB initiator.

Mechanistic studies have shown that the quenching of ^3^O_2_ by ^3^MB^+*^ is the main deoxygenation pathway in the Cu/MB^+^ dual-catalytic system. This was further supported by the observation that the percentage of new chains generated during ATRP is low, even at high DP_T_ (Table 2). The formation of ^3^MB^+*^ and the continuous regeneration of the [Cu^I^/L]^+^ activator by MB^•^ allow for effective oxygen scavenging and photo-ATRP to be performed in the open air.

### Polymerization under Different Light Wavelengths

**Figure 5:**
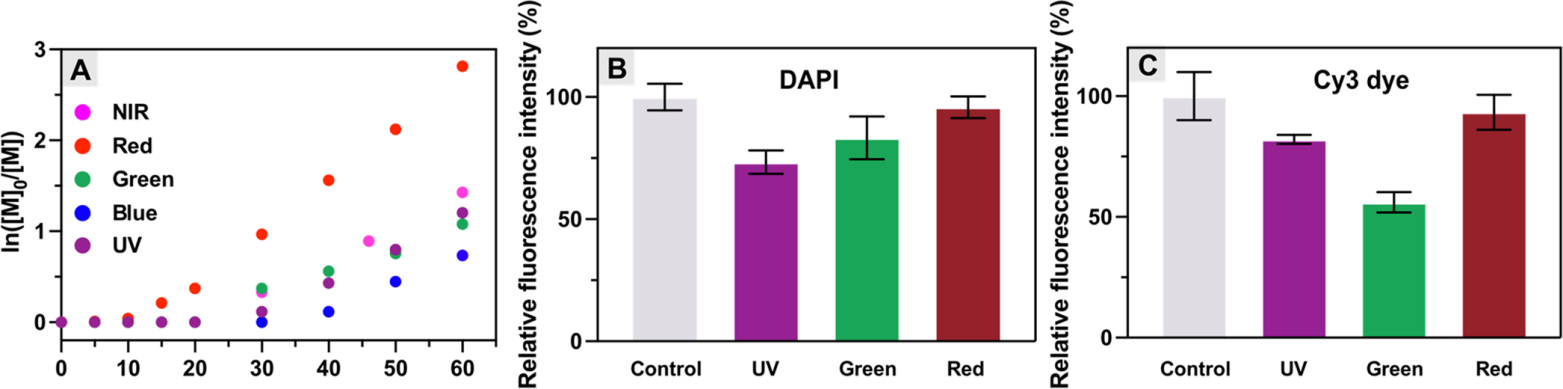
(A) Kinetic plots for MB^+^/Cu-catalyzed photo-ATRP of OEOMA_500_ under different light wavelengths and photobleaching of (B) DAPI and (C) Cy3 dye in 1× PBS under irradiation of different lights for 30 min.

Although visible light with wavelengths in the range of 400–750 nm accounts for half of the solar energy reaching the surface of Earth, there are very few effective PCs that can harness this energy for polymer materials synthesis.^15,85^ The broad absorption of MB^+^ observed (Figure S12) prompted us to investigate the efficiency of the dual-catalytic system under irradiation with a wide range of visible light, including UV, blue, green, red, and NIR light (Table 3). In all cases, high monomer conversions (50–90%) and low dispersity values (1.16 < *Đ* < 1.25) were achieved within 60 min. Moreover, essentially linear semilogarithmic kinetic plots were observed after variable induction periods for different light sources (Figure 5A). Finally, rapid polymerization was observed in the presence of sunlight, highlighting the great potential of the MB^+^/Cu catalytic system as a green and sustainable approach to polymer synthesis.

**Table 3:** Polymerization of OEOMA_500_ Using Different Light Wavelengths^a^

entry	light	λ_max_ (nm)	intensity (mW cm^–2^)	conv. (%)^b^	*M*_n,th_	*M*_n,app_^c^	*M*_n,MALS_^d^	*Đ*^c^
1	UV	395	30	70	70 000	40 600	71 700	1.16
2	blue	445	45	52	52 000	33 600	58 900	1.18
3	green	527	20	66	66 000	41 600	75 900	1.17
4	red	630	25	90	90 000	54 000	94 100	1.21
5	NIR	730	55	76	76 000	41 200	82 600	1.25
6	sunlight			90	90 000	56 400	110 700	1.22

a Reaction conditions: [OEOMA_500_]/[HO-EBiB]/[MB^+^]/[CuBr_2_]/[TPMA] = 200/1/0.025/0.3/0.9, [OEOMA_500_] = 300 mM, in 1× PBS with DMSO (10% v/v), irradiated for 60 min under different LEDs in an open-air 96-well plate at a volume of 250 μL.

b Monomer conversion was determined by using ^1^H NMR spectroscopy.

c Molecular weight (*M*_n,app_) and dispersity (*Đ*) were determined by SEC analysis (DMF as eluent) calibrated to poly(methyl methacrylate) standards.

d Absolute molecular weight (*M*_n,MALS_) was determined by SEC analysis (DMF as eluent) with a multiangle light scattering (MALS) detector.

### Polymerization at Different Volumes and Light Intensities

Low-volume open-air RDRP has broad and practical applications, particularly in high-throughput screening and bioconjugate synthesis.^87,92^ When the reaction volume was reduced from 250 to 100 μL in an open-air 96-well plate, the monomer conversion remained in the range 50–70% after 30 min (Table S3, entries 2–5). However, further reduction of the volume to 50 μL resulted in a significant drop in conversion to 6% (Table S3, entry 1). This could be attributed to increased oxygen diffusion into the polymerization solution at a low volume. Using a micropipet tip as the reaction vessel for the same volume (50 μL) resulted in an increase in monomer conversion (31%) due to the smaller surface-to-volume ratio and decreased oxygen diffusion. In addition, the polymer had a low dispersity of 1.19 (Table S3, entry 6).

Large-scale reactions at 1.8 and 4.5 mL were also successfully carried out, achieving approximately 50% conversion with dispersity values below 1.20 after 60 min (Table S3, entries 7, 8). Finally, higher light intensities significantly improved the polymerization rate but at the expense of control over the ATRP (Table S4).

### Orthogonality of MB^+^/Cu-Catalyzed Photo-ATRP

Performing photo-RDRP at the desired wavelength provides the opportunity to synthesize light-absorbing polymers or biohybrids labeled with molecules/dyes susceptible to photobleaching.^121^ As illustrated in Figure 5B and C, the most common dyes undergo photobleaching when irradiated with a specific wavelength. The developed full-spectrum MB^+^/Cu dual-catalytic system not only allows the synthesis of well-defined polymers but also minimizes photobleaching by selecting the most suitable light wavelength for photo-ATRP in the presence of dye-labeled substrates. For example, 4,6-diamidino-2-phenylindole (DAPI) shows strong photobleaching under UV and also under green light irradiation after only 30 min, while its fluorescence intensity remains unchanged under red light (Figure 5B). A similar photobleaching phenomenon was observed for another commonly used cyanine dye (Cy3, Figure 5C), especially under green light (527 nm). Consequently, MB^+^/Cu-catalyzed photo-ATRP can be performed under different light wavelengths to modify dye-containing (bio)macromolecules, effectively avoiding photobleaching.

### Monomer Scope

We were encouraged to extend the monomer scope of this system for the synthesis of a library of functional polymers with different charge (neutral, anionic, cationic, and zwitterionic) and solubility (hydrophilic and hydrophobic) by polymerizing methacrylate, acrylate, and acrylamide monomers at fixed molar ratios of [M]/[HO-EBiB]/[MB^+^]/[CuBr_2_]/[L] = 200/1/0.025/0.3/0.9 (Table 4). All monomers were polymerized under red light irradiation in a controlled manner to form polymers with low dispersity values (*Đ* < 1.27).

**Table 4:**
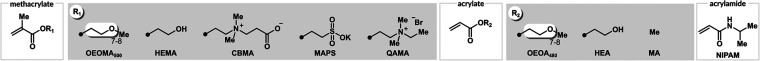
Polymerization of Various Monomers^a^

entry	monomer (M)	ligand	conv. (%)^b^	*M*_n,th_	*M*_n,app_^c^	*Đ*^c^
1	OEOMA_500_	TPMA	62	62 000	48 900	1.25
2	NIPAM	Me_6_TREN	77	17 400	25 000	1.19
3	OEOA_480_	Me_6_TREN	77	67 200	51 000	1.23
4	CBMA	TPMA	48	22 000	36 800	1.19
5	QAMA	TPMA	65	34 600	23 700	1.27
6	MAPS	TPMA	92	45 300	43 400	1.25
7	HEA	Me_6_TREN	60	13 900	26 800	1.19
8^d^	MA	Me_6_TREN	90	15 700	16 200^e^	1.09^e^
9^d^	HEMA	TPMA	43	11 400	15 500	1.27

a Reaction conditions: [M]/[HO-EBiB]/[MB^+^]/[CuBr_2_]/[L] = 200/1/0.025/0.3/0.9, [OEOMA_500_] = 300 mM, in 1× PBS with DMSO (10% v/v), irradiated for 30 min under red LEDs (630 nm, 25 mW cm^–2^) in an open-air 96-well plate at a volume of 250 μL.

b Monomer conversion was determined by using ^1^H NMR spectroscopy.

c Molecular weight (*M*_n,app_) and dispersity (*Đ*) were determined by SEC analysis (DMF as eluent) calibrated to poly(methyl methacrylate) standards.

d Reaction conditions: [M]/[EBiB]/[MB^+^]/[CuBr_2_]/[L] = 200/1/0.005/0.2/1.2, [M] = 5.5 M, in DMSO, irradiated for 60 min under red LEDs (630 nm, 2.5 mW cm^–2^).

e Molecular weight (*M*_n,app_) and dispersity (*Đ*) were determined by SEC analysis (THF as eluent) calibrated to poly(methyl methacrylate) standard.

In addition, the MB^+^/Cu dual-catalytic system was also used for the polymerization of the hydrophobic monomer, methyl acrylate (MA), in organic solvent (DMSO) using ethyl α-bromoisobutyrate (EBiB) as the initiator and Me_6_TREN (Me_6_TREN = tris[2-(dimethylamino)ethyl]amine) as the ligand (Table S5). A high monomer conversion of 90% was obtained in 30 min, yielding a polymer with low dispersity (*Đ* = 1.09) and excellent control over MW (*M*_n,th_ = 15 700, *M*_n,app_= 16 200, Table 4, entry 8).

### Synthesis of DNA–Polymer Bioconjugates

**Figure 6:**
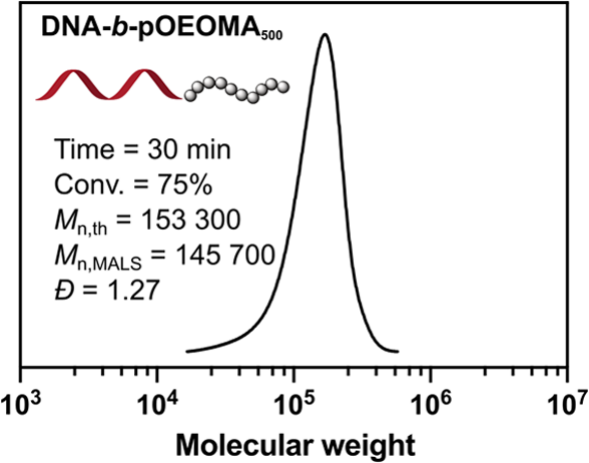
Synthesis of the DNA–polymer bioconjugate.

Aqueous photo-RDRP can be used to prepare well-defined bioconjugates for biomedical applications.^51,122,123^ Since nucleic acids have negligible absorption in the red light range, we attempted to synthesize DNA-based bioconjugates using MB^+^/Cu-catalyzed photo-ATRP. To test the feasibility of this mild dual-catalytic system in the synthesis of biohybrids, we prepared a DNA–polymer bioconjugate by the “grafting-from” approach (Figure 6).^123−127^ DNA functionalized with a single ATRP initiator at the 5′ terminal (T_10_-Br) was synthesized as previously reported^31,127^ and then used for chain extension with the hydrophilic OEOMA_500_ under the optimized conditions using the molar ratio [OEOMA_500_]/[T_10_-Br]/[MB^+^]/[CuBr_2_]/[TPMA] = 400/1/0.05/0.6/1.8. After 30 min of red light irradiation, a well-defined bioconjugate (DNA-*b*-pOEOMA_500_) was obtained with 76% monomer conversion, excellent correlation between *M*_n,th_ and *M*_n,MALS_, and low dispersity (*Đ* = 1.27). This result highlights the potential of MB^+^/Cu-catalyzed photo-ATRP for the precise synthesis of DNA–polymer bioconjugates.^124^

### Biocompatibility of MB^+^/Cu Dual-Catalytic Systems

**Figure 7:**
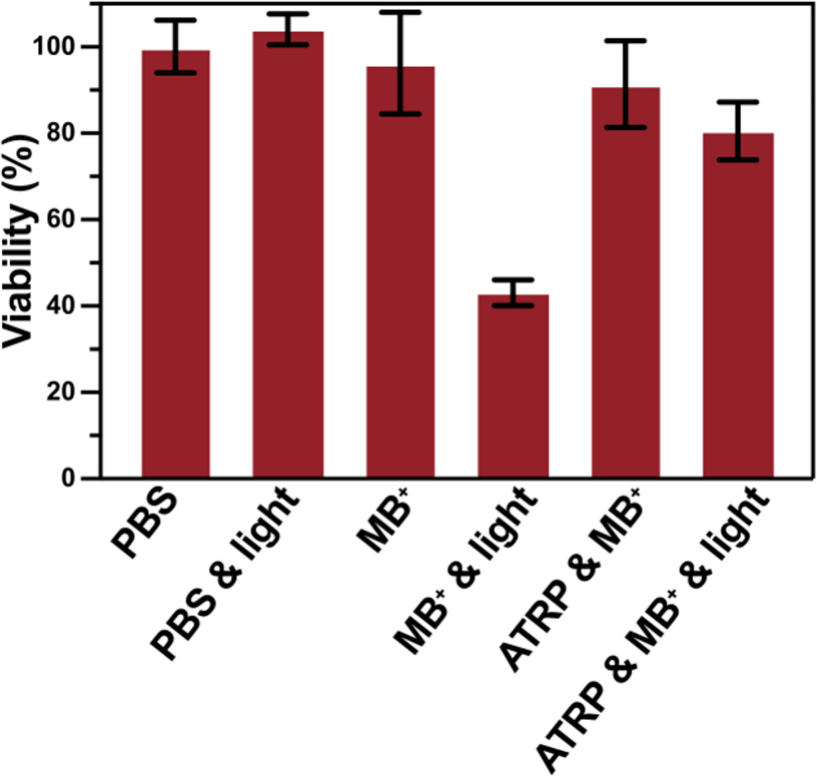
Biocompatibility of red-light-induced ATRP in the presence of cells. Control group: 1× PBS without light irradiation. Quantification of cell viability by fluorescence intensities measured with a TECAN spectrophotometer reader. Bars indicate mean ± SEM (*n* = 6); conditions for MB^+^: [MB^+^] = 0.01 mM, in PBS with DMSO (0.27% v/v); reaction conditions for ATRP: [OEOMA_500_]/[HO-EBiB]/[MB^+^]/[CuBr_2_]/[TPMA] = 200/1/0.00067/0.05/0.6; [OEOMA_500_] = 300 mM, in 1× PBS with DMSO (0.27% v/v), irradiated for 5 min under red light LEDs (λ_max_ = 630 nm, 25 mW cm^–2^) in 96-well plates.

The biocompatibility of MB^+^ and the mild reaction conditions suggest that our method could be used to synthesize well-defined polymers in the presence of cells.^128−130^ The cytocompatibility of the MB^+^/Cu dual-catalytic system was investigated by polymerization in the presence of a fibroblast cell line (NIH3T3 cells). When the control group was exposed to red light, negligible cell death was observed (Figure 7). Similarly, in the presence of an MB^+^ (0.01 mM) and ATRP “cocktail”, only around 4% and 9% decreases in cell viability were observed, respectively. However, turning on the light for 5 min in the presence of MB^+^ alone caused ∼60% cell death. This high toxicity can be attributed to the rapid binding and internalization of the positively charged methylene blue into the cells and the subsequent damage caused by the singlet oxygen or free radicals.^131^ Surprisingly, after 5 min of red light irradiation, the ATRP reaction mixture showed ∼80% cell viability, indicating the good biocompatibility of MB^+^/Cu-catalyzed photo-ATRP. This improved biocompatibility of the MB^+^/Cu dual-catalytic system is plausibly due to the effective quenching of the excited MB^+^ and MB^•^ by the [Br–Cu^II^/TPMA]^+^ complex. It will be interesting to further explore the applicability of this methodology in the modification of cells.

## Conclusions

We have demonstrated the first example of fully oxygen-tolerant photo-ATRP under red/NIR light irradiation using the commercially available and biocompatible photocatalyst methylene blue. Mechanistic studies confirmed that the TPMA ligand used in excess bound to copper cations and served as a sacrificial electron donor, reductively quenching ^3^MB^+^*. This resulted in the formation of the MB radical (MB^•^) and an amine radical cation. Subsequently, MB^•^ reduced [X–Cu^II^/L]^+^ by single electron transfer, generating the [Cu^I^/L]^+^ activator and the MB^+^ photocatalyst in its ground state. The formation of ^3^MB^+^* and the continuous regeneration of the [Cu^I^/L]^+^ activator by MB^•^ allowed effective oxygen scavenging and photo-ATRP to be performed in open air. Control over radical propagation was achieved by a reversible redox equilibrium between Cu^I^/Cu^II^ complexes, where they intermittently activated dormant species and deactivated radicals. MB^+^ and an excess of ligand were essential to induce and maintain photo-ATRP in the presence of oxygen.

The MB^+^/Cu dual-catalytic system allowed obtaining well-defined polymers with narrow molecular weight distributions (*Đ* < 1.20) in an open-air 96-well plate. The high-throughput screening enabled rapid optimization of the polymerization conditions, the synthesis of polymers with varying degrees of polymerization (DP_T_ = 50–1500), and polymerization of several functional acrylates, methacrylates, and acrylamides. In addition, the broad absorption spectrum of MB^+^ allowed polymerization under UV to NIR irradiation (395–730 nm), which opens avenues for the integration of orthogonal photoinduced reactions. As an example, a DNA–polymer bioconjugate was synthesized in a controlled manner. Finally, polymerization performed in a cellular environment resulted in good cell viability, confirming the biocompatibility of the proposed methodology.

## Supporting Information Available

Details of experimental procedures and additional experiments and polymerization results (PDF)
