# Supplementary material for: Red-Light-Driven Atom Transfer Radical Polymerization for High-Throughput Polymer Synthesis in Open Air
Source: J Am Chem Soc. 2023 Oct 25;145(44):24315–27. doi: 10.1021/jacs.3c09181 (PMC10636753; doi:10.1021/jacs.3c09181)
Supplement: Supplementary file 1 — ja3c09181_si_001.pdf [file ja3c09181_si_001.pdf]

Supporting Information

Red-Light-Driven Atom Transfer Radical Polymerization for High-Throughput Polymer Synthesis In Open Air

Xiaolei Hu,[1] Grzegorz Szczepaniak,*[1,2] Anna Lewandowska-Andralojc,[3,4] Jaepil Jeong,[1,5] Bingda Li,[6] Hironobu Murata,[1] Rongguan Yin,[1] Arman Moini Jazani,[1] Subha R. Das,[1,5] and Krzysztof Matyjaszewski*[1]

[1]Department of Chemistry, Carnegie Mellon University, Pittsburgh, Pennsylvania 15213, USA

[2]University of Warsaw, Faculty of Chemistry, Pasteura 1, 02-093 Warsaw, Poland

[3]Faculty of Chemistry, Adam Mickiewicz University, Uniwersytetu Poznanskiego 8, 61-614 Poznan, Poland

[4]Center for Advanced Technology, Adam Mickiewicz University, Uniwersytetu Poznanskiego 10, 61-614 Poznan, Poland

[5]Center for Nucleic Acids Science & Technology, Carnegie Mellon University, Pittsburgh, Pennsylvania 15213, USA

[6]Department of Biomedical Engineering, Carnegie Mellon University, Pittsburgh, Pennsylvania 15213, USA

Contents

Experimental *3*
Materials 3
Instrumentation *3*
Photoreactor for MB^+/Cu-catalyzed photo-ATRP 3
^{1}H Nuclear magnetic resonance (^{1}H NMR) 4
Size exclusion chromatography (SEC) 4
Size exclusion chromatography with a multi-angle light scattering (SEC-MALS) 4
Microplate reader 4
DNA synthesis 5
Procedures *5*
General procedure for MB^+/Cu-catalyzed photo-ATRP of $OEOMA_{500}$ under red light irradiation 5
Kinetic study (Figure 2) 7
Synthesis of $pOEOMA_{500}$ with varying DP_T (Figure 3A) 11
Chain extension (Figure 3B and S5) 11
Temporal control (Figure 3C) 13
Mechanistic study (Figure 4) 14
Calculation of Gibbs free energy for the photoinduced electron transfer 20
Polymerization under different light wavelengths (Figure 5A) 22
Polymerization at varying scale 22
Polymerization at different light intensity 23
Photobleaching of common dyes under different light (Figure 5B and C) 23
Polymerization in DMSO 24
Synthesis of DNA-polymer bioconjugate (Figure 6) 24
Biocompatibility of MB^+/Cu-catalyzed photo-ATRP in presence of cells (Figure 7) 25
References *27*

Experimental

Materials

Unless otherwise noted, all chemicals were purchased from commercial sources and used as received. Methylene blue (MB^+, 99%), copper(II) bromide ($CuBr_2$, 99.99%), 2-hydroxyethyl α-bromoisobutyrate (HO-EBiB, 95%), oligo(ethylene glycol) methyl ether methacrylate (average M_n = 500, $OEOMA_{500}$), oligo(ethylene glycol) methyl ether acrylate (average M_n = 480, $OEOA_{480}$), *N*-isopropylacrylamide (NIPAM, purified by recrystallization in hexane), 3-sulfopropyl methacrylate potassium salt (MAPS), 2-hydroxyethyl acrylate (HEA), 2-hydroxyethyl methacrylate (HEMA), methyl acrylate (MA), ethyl α-bromoisobutyrate (EBiB, ≥99%), SnatchCat (quenching agent), were purchased from *Sigma-Aldrich*. All monomers were passed through a column of basic alumina to remove the inhibitor prior to the use tris(2-pyridylmethyl)amine (TPMA, 99%), tris[2-(dimethylamino)ethyl]amine (Me_6TREN, 99%) were purchased from *AmBeed*. 10X phosphate-buffered saline (10X PBS), 1X dulbecco's phosphate-buffered saline (DPBS), 4′,6-diamidino-2-phenylindole (DAPI), and LIVE/DEAD™ Viability/Cytotoxicity Kit were purchased from *Thermo Fisher Scientific*. Water (HPLC grade), dimethylformamide (DMF, HPLC grade), tetrahydrofuran (THF, HPLC grade), and dimethyl sulfoxide (DMSO, HPLC grade) were purchased from *Fisher Chemical*. D_2O and DMSO-d_6 were purchased from *Cambridge Isotope Laboratories, Inc.* Deoxyribonucleotide (DNA) phosphoramidites and CPG solid support for DNA synthesis were purchased from *Chemgenes* and *Glen Research*. Cy3-alkyne was purchased from *Lumiprobe*. Quaternary ammonium monomer 2-(dimethylethylammonium)ethyl methacrylate (QAMA) was synthesized according to literature procedure.[1]

Instrumentation

Photoreactor for MB^+/Cu-catalyzed photo-ATRP

Polymerizations were conducted in a 96-well plate mounted on a 96-point LED array with UV light (395 nm, 30 mW cm^{-2}), blue light (445 nm, 45 mW cm^{-2}), green light (527 nm, 20 mW cm^{-2}), red light (630 nm, mW cm^{-2}), and NIR light (730 nm, mW cm^{-2}), and Lumidox

Gen II LED Controller, which allows controlling reaction time and light intensity, were purchased from *Analytical Sales and Services, Inc.* Photo-ATRP of MA in DMSO was carried out in a glass insert under red LEDs (630 nm, 2.5 mW cm^{-2}). The red LED strips (*aspectLED*) were mounted inside a glass container (diameter = 9 cm, height = 7 cm).

^{1}H Nuclear magnetic resonance (^{1}H NMR)

^{1}H NMR spectra were recorded on *Bruker Avance III* 500 MHz spectrometer with D_2O or DMSO-d_6 used as the solvent.

Size exclusion chromatography (SEC)

SEC measurements of $pOEOMA_{500}$, $pOEOA_{480}$, pNIPAM, pHEMA, and pHEA were performed using *PSS* columns (Styrogel 10^5, 10^3, 10^2 Å) with DMF containing LiBr (0.05 M) as the eluent at 50 °C and the flow rate of 1 mL/min. SEC measurements of pMA were conducted using *PSS* columns (Styrogel 10^2, 10^3, 10^4, 10^5 Å) with THF as the eluent at 35 °C and the flow rate of 1 mL/min. Linear poly(methyl methacrylate) standards were used for SEC calibration. Absolute molecular weight ($M_{n,MALS}$) was determined by SEC analysis (DMF as eluent) with a multi-angle light scattering (MALS) detector.

Size exclusion chromatography with a multi-angle light scattering (SEC-MALS)

SEC-MALS measurements of cationic, anionic and zwitterionic polymers were conducted using the Agilent SEC system (*Agilent*, 1260 Infinity II with UV detector) coupled with viscometer, MALS, DLS, and RI detectors (*Wyatt Technology*, USA). Measurements were performed using the Waters Ultra hydrogel linear column with 1X DPBS as an eluent at the flow rate of 0.5 mL/min and at room temperature.

Microplate reader

The fluorescence intensity of dyes was recorded by using the microplate reader (The Infinite® M1000, *Tecan*).

DNA synthesis

The model DNA initiator (T_{10}-Br) was synthesized following the previously reported protocol using the MerMade 4 oligonucleotide synthesizer (*Bioautomation*).[2]

Procedures

General procedure for MB^+/Cu-catalyzed photo-ATRP of $OEOMA_{500}$ under red light irradiation

First, stock solutions of $OEOMA_{500}$ (600 mM in H_2O), MB^+ (1.875 mM in H_2O), HO-EBiB (75 mM in DMSO), $CuBr_2$ (11.25 mM in H_2O), and TPMA (67.49 mM in DMSO) were prepared (Figure S2A). A typical ATRP "cocktail" solution (250 µL) was then prepared as follows. $OEOMA_{500}$ stock (125 µL) MB^+ stock (5 µL), $CuBr_2$ stock (10 µL), TPMA stock (5 µL), HO-EBiB stock (5 µL), DMSO (15 µL), H_2O (60 µL) and 10X PBS solution (25 µL) were then mixed (Figure S2B). The final concentrations were $OEOMA_{500}$ (300 mM), MB^+ (37.5 µM), $CuBr_2$ (0.45 mM), TPMA (1.35 mM), HO-EBiB (1.5 mM), DMSO (10% *v*/*v*), and 1X PBS. The ATRP "cocktail" was then transferred to a 96-well plate (Figure S2C-E). The 96 well plate with ATRP "cocktail" solutions was mounted on a Lumidox® Gen II 96-point LED array. The polymerization mixtures were irradiated under red light LEDs (630 nm, 25 mW cm^{-2}) for 30 min (Figure S2E). Samples were taken for ^1H NMR and SEC characterization.

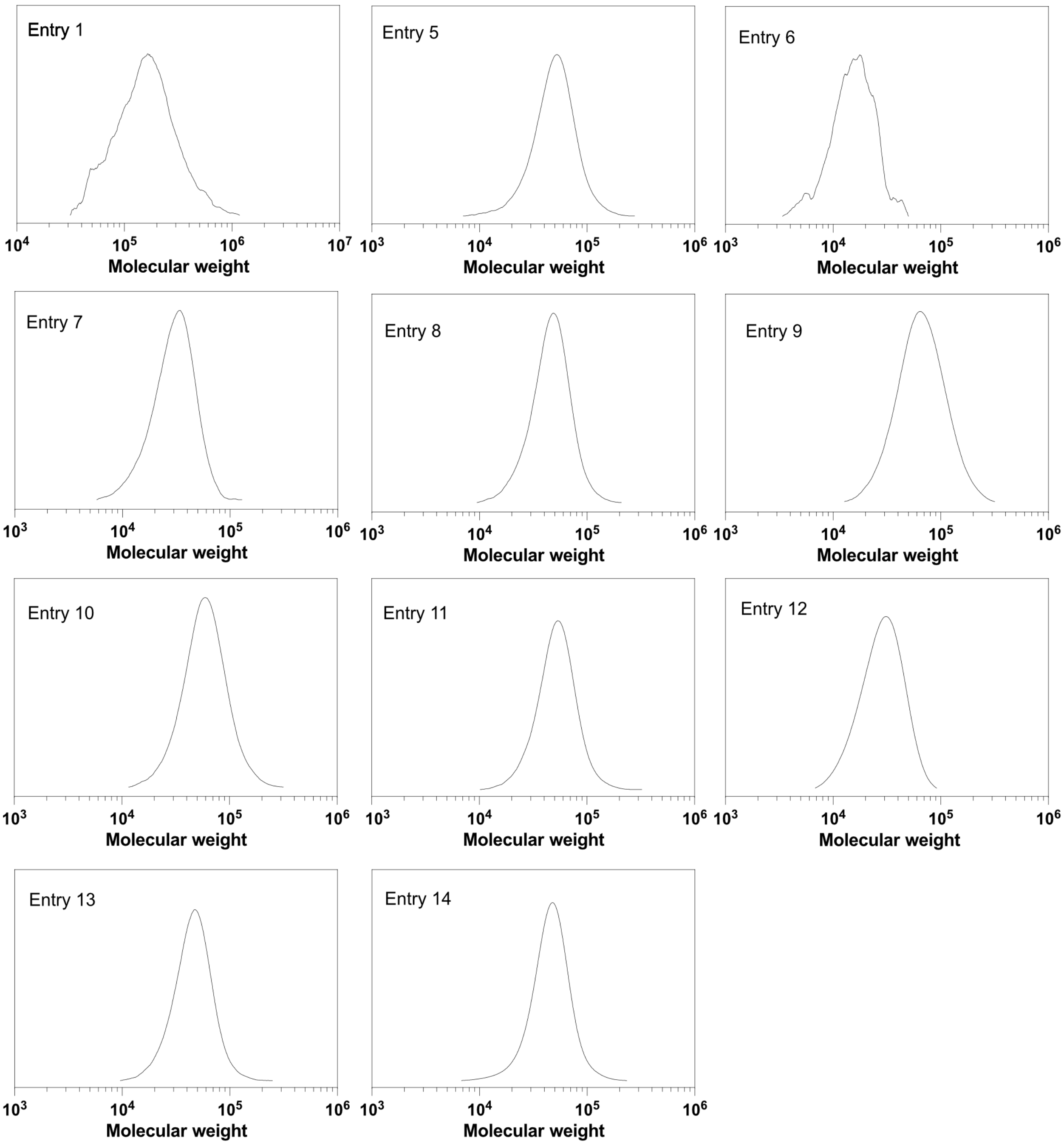

Figure S1. SEC traces for the polymerizations shown in Table 1.

Kinetic study (Figure 2)

As a typical example, an ATRP “cocktail” solution (5 mL) was prepared based on the general procedure for MB^+/Cu-catalyzed photo-ATRP with final concentrations of $OEOMA_{500}$ (300 mM), MB^+ (37.5 µM), $CuBr_2$ (0.45 mM), TPMA (1.35 mM), HO-EBiB (1.5 mM), DMSO (10% *v*/*v*), and 1X PBS. The solution was first transferred to a multichannel pipette reservoir (20 mL), and then 250 µL of solution was disposed to each well in a 96-well plate using a multichannel pipette (12-channel). The 96-well plate was mounted on a Lumidox® Gen II 96-point LED array. Polymerization mixtures were irradiated for 60 min under red light (630 nm, 25 mW cm^{-2}). At different time intervals (0, 5, 10, 15, 20, 30, 40, 50, and 60 min), SnatchCat solution (50 µl, 1 mg/mL in H_2O) was added to the corresponding wells to quench the polymerizations.[3] At the end of the polymerizations, all samples were withdrawn and analyzed by ^1H NMR and SEC techniques.

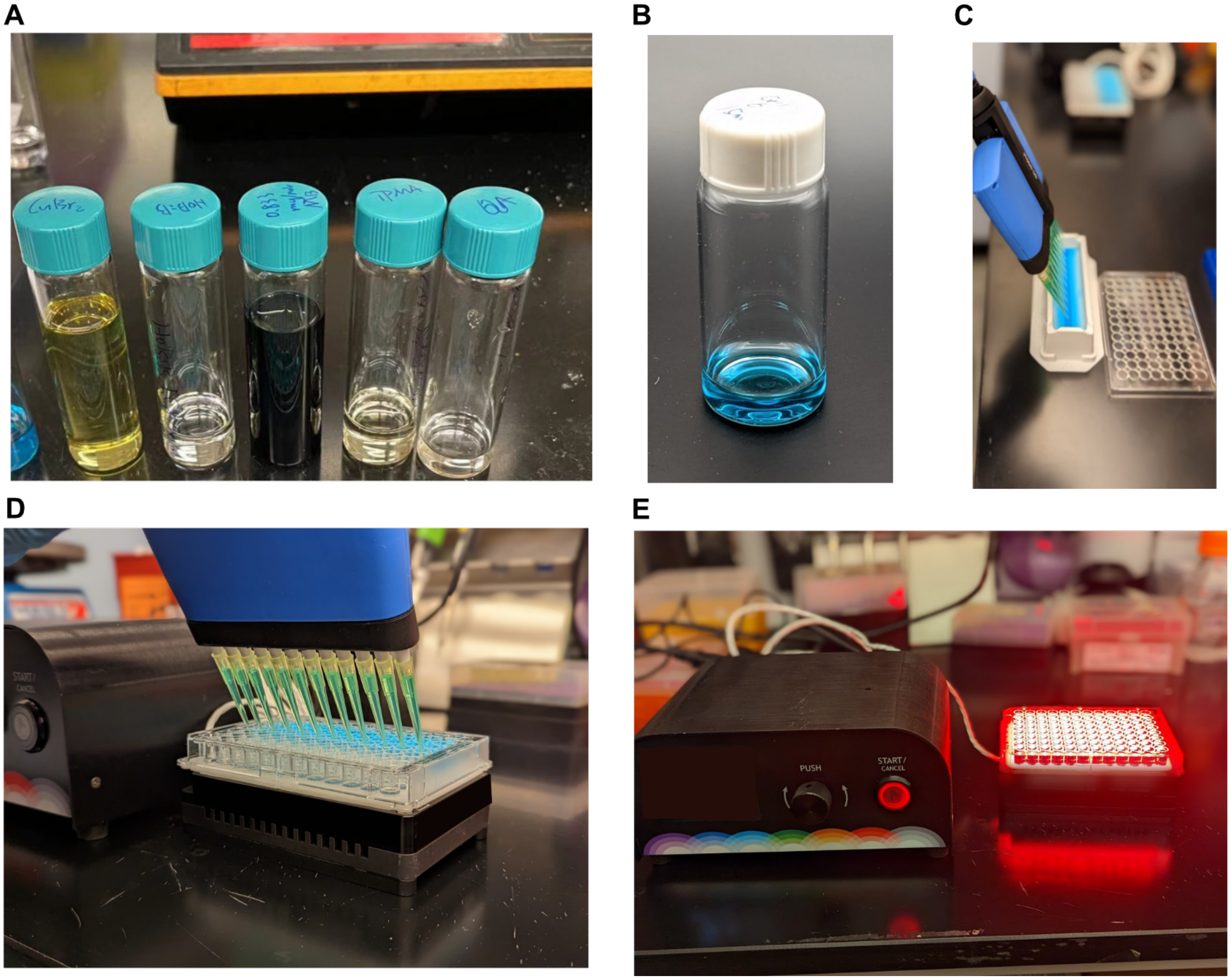

Figure S2. High-throughput set-up for MB^+/Cu-catalyzed photo-ATRP; stock solutions of $OEOMA_{500}$, MB^+, HO-EBiB, $CuBr_2$, and TPMA (A), ATRP “cocktail” in a vial (B), multichannel pipette reservoir (C), 96-well plate (D), and on a Lumidox® Gen II 96-point LED array (E).

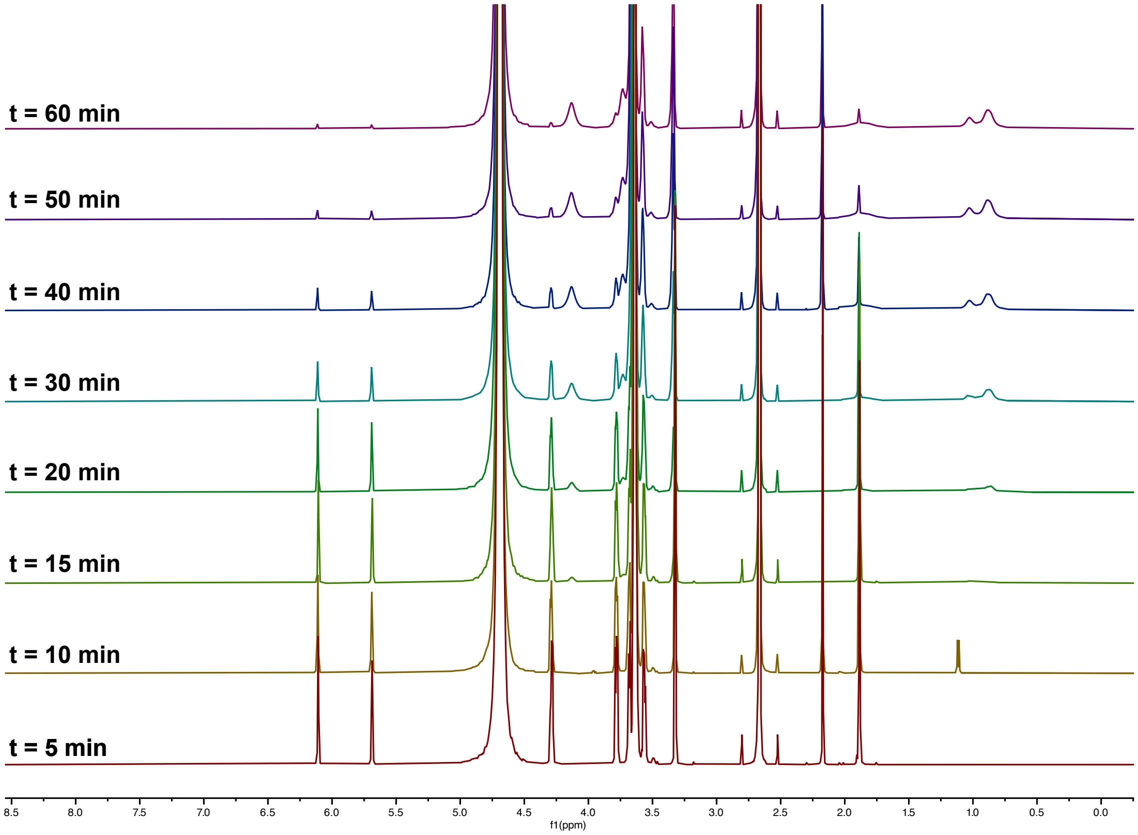

Figure S3. ^{1}H NMR for the samples taken at varying polymerization times during the kinetic study.

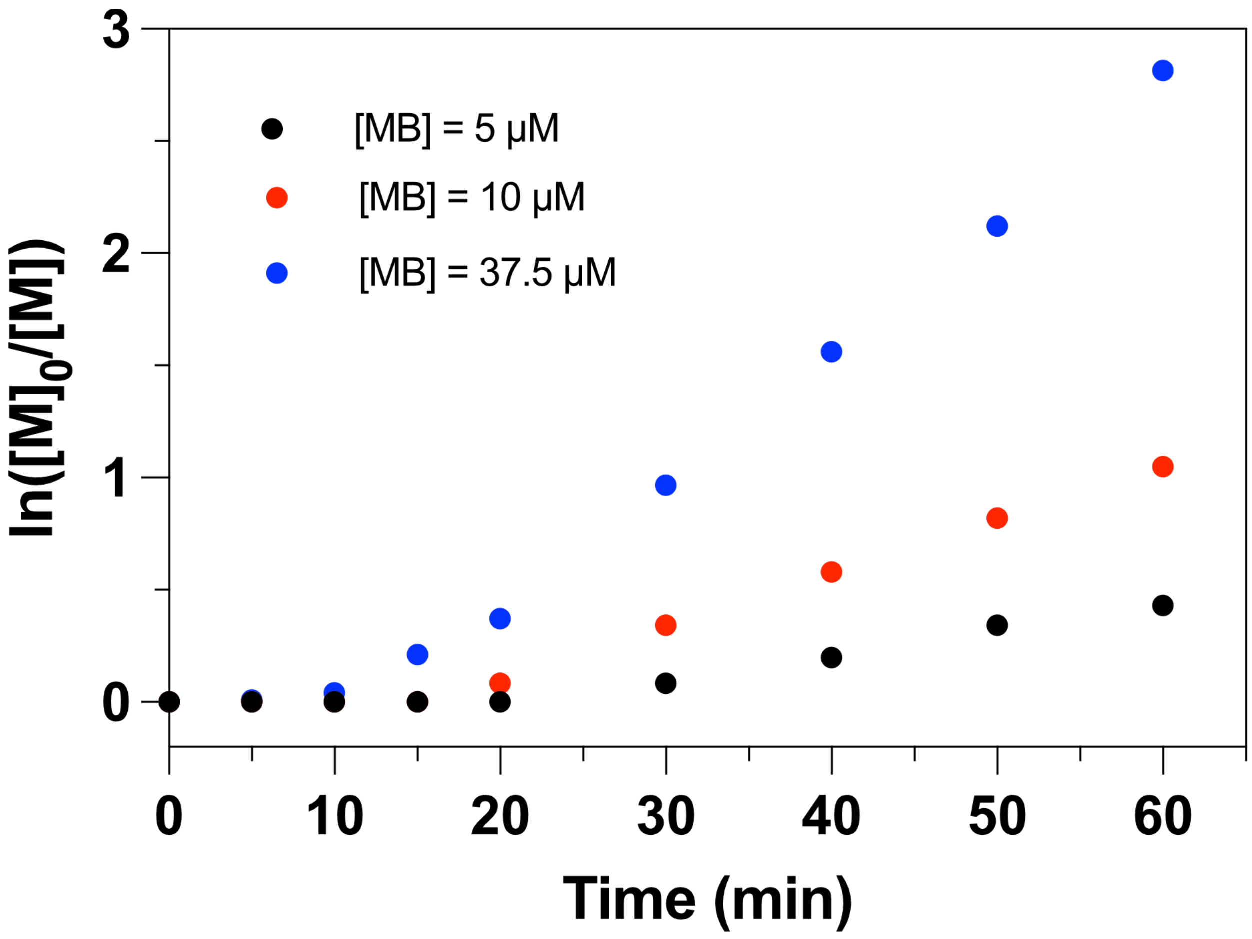

Figure S4. Kinetic study of photo-ATRP at varying MB^+ concentration. Reaction conditions: $[OEOMA_{500}]/[HO\text{-}EBiB]/[MB^+]/[CuBr_2]/[TPMA]$ = 200/1/X/0.3/0.9, $[OEOMA_{500}]$ = 300 mM, in PBS with DMSO (10% *v*/*v*), under irradiation of red LEDs (630 nm, 25 mW cm^{-2}).

Synthesis of $pOEOMA_{500}$ with varying DP_T (Figure 3A)

The target degree of polymerization (DP_T = 50, 100, 200, 400, 600, 800, 1000, and 1500) was varied by adjusting the HO-EBiB initiator concentration ([HO-EBiB] = 6.0, 3.0, 1.5, 0.75, 0.5, 0.375, 0.3, and 0.2 mM) while keeping the other components at the same concentration: $OEOMA_{500}$ (300 mM), MB^+ (37.5 μM), $CuBr_2$ (0.45 mM), and TPMA (1.35 mM). ATRP "cocktail" solutions were prepared based on the general procedure and transferred to a 96-well plate. The polymerization mixtures were irradiated for 30 min under red LEDs (630 nm, 25 mW cm^{-2}). At the end of the polymerizations, all samples were withdrawn and analyzed by ^1H NMR and SEC techniques.

Table S1. Polymerization of $OEOMA_{500}$ with varying DP_T by tuning monomer concentration.[a]

Entry	DP_T	$[OEOMA_{500}]$ (mM)	Conv. (%)	$M_{n,th}$	$M_{n,app}$	$M_{n,MALS}$	$Đ$
1	100	150	54	27 000	21 700	42 200	1.19
2	150	225	68	51 000	33 700	61 940	1.19

[a]Reaction conditions: $[OEOMA_{500}]/[HO\text{-}EBiB]/[MB^+]/[CuBr_2]/[TPMA]$ = x/1/0.025/0.3/0.9 (x = 100, 150 and 200 by tuning monomer concentration), [HO-EBiB] = 1.5 mM, in 1X PBS with DMSO (10% *v*/*v*), irradiated for 30 min under red LEDs (630 nm, 25 mW cm^{-2}) in a 96-well plate. Molecular weight ($M_{n,app}$) and dispersity ($Đ$) were determined by SEC analysis (DMF as eluent) calibrated to poly(methyl methacrylate) standards. Absolute molecular weight ($M_{n,MALS}$) was determined by SEC analysis (DMF as eluent) with a multi-angle light scattering (MALS) detector.

Chain extension (Figure 3B and S5)

For the synthesis of $pOEOMA_{500}$-*b*-$pOEOMA_{500}$ block copolymer, $pOEOMA_{500}$ with DP_T = 50 was first synthesized under the condition of $[OEOMA_{500}]/[HO\text{-}EBiB]/[MB^+]/[CuBr_2]/[TPMA]$ = 50/1/0.025/0.3/0.9 and irradiation of red LEDs (630 nm, 25 mW cm^{-2}) for 30 min in a 96-well plate. Subsequently, the crude macroinitiator sample $pOEOMA_{500}$ taken from the post-polymerization solution was used directly after

polymerization to prepare the ATRP "cocktail" for chain extension with $OEOMA_{500}$ at DP_T = 200 under the condition of $[OEOMA_{500}]/[pOEOMA_{500}]/[MB^+]/[CuBr_2]/[TPMA]$ = 200/1/0.025/0.3/0.9 in 1X PBS with DMSO (10% *v*/*v*). The polymerization mixture was irradiated under red LEDs (630 nm, 25 mW cm^{-2}) for 30 min. Finally, the sample was withdrawn and analyzed by ^{1}H NMR and SEC techniques.

For $pOEOMA_{500}$-*b*-pNIPAAm block copolymer, $pOEOMA_{500}$ with DP_T = 200 was first synthesized under the condition of $[OEOMA_{500}]/[HO\text{-}EBiB]/[MB^+]/[CuBr_2]/[TPMA]$ = 200/1/0.025/0.3/0.9. Crude $pOEOMA_{500}$ was purified by dialysis with H_2O, and then used as the macro-initiator to prepare an ATRP "cocktail" for chain extension with NIPAM at DP_T = 200 under $[NIPAM]/[pOEOMA_{500}]/[MB^+]/[CuBr_2]/[Me_6TREN]$ = 200/1/0.025/0.3/0.9 in 1X PBS with DMSO (10% *v*/*v*). The polymerization mixture was irradiated under red LEDs (630 nm, 25 mW cm^{-2}) for 30 min. Finally, the samples were withdrawn and analyzed by ^{1}H NMR and SEC techniques.

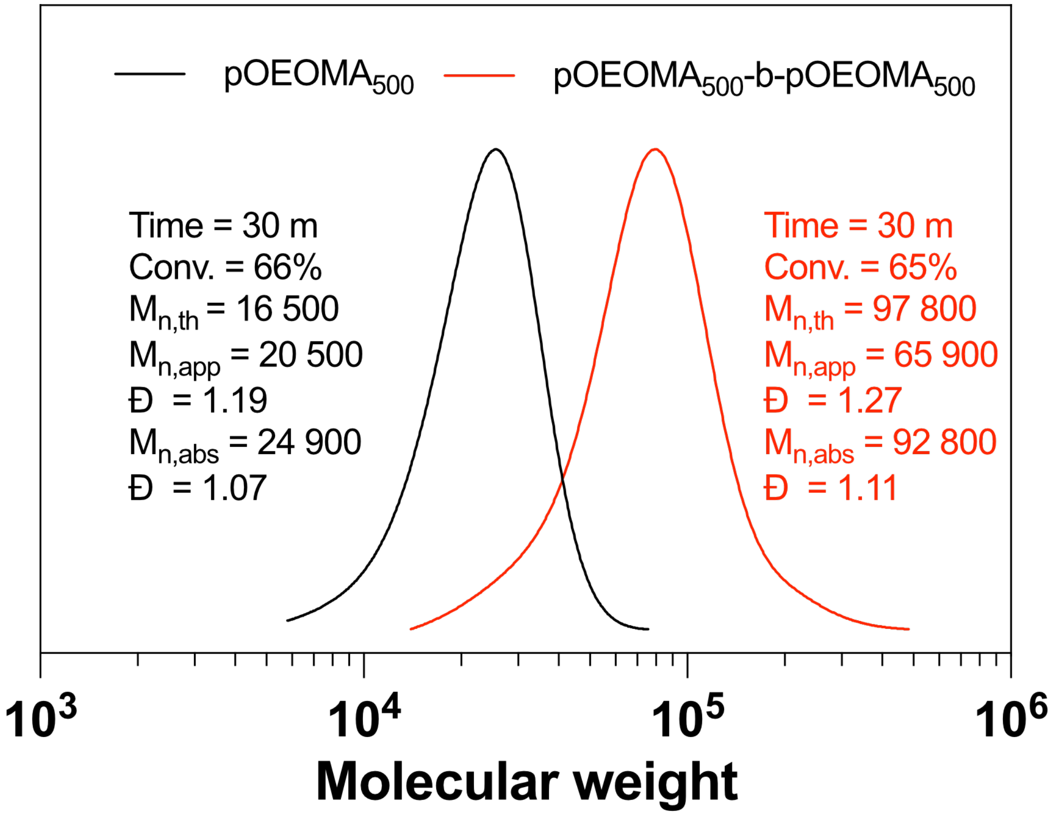

Figure S5. Chain extension of $pOEOMA_{500}$ macro-initiator with $OEOMA_{500}$ monomer.

Temporal control (Figure 3C)

The ATRP "cocktail" solution (5 mL) was prepared based according to the general procedure for MB^+/Cu-catalyzed photo-ATRP at concentrations of $OEOMA_{500}$ (300 mM), MB^+ (37.5 μM), $CuBr_2$ (0.45 mM), TPMA (1.35 mM), HO-EBiB (1.5 mM), DMSO (10% *v*/*v*), and 1X PBS. The solution was first transferred to a multichannel pipette reservoir (20 mL), and then to each well in a 96-well plate using a multichannel pipette. The ATRP "cocktail" solutions in a 96-well plate were irradiated for 60 min under red light LEDs (630 nm, 25 mW cm^{-2}). At different time intervals (0, 15, 30, 45, 60, and 75 min), SnatchCat solution (50 μl, 1 mg/mL in H_2O) was added to the corresponding wells to stop the polymerizations.[3] At the end of the polymerizations, all samples were withdrawn and analyzed by ^1H NMR and SEC techniques. The light was switched on/off periodically together with adding of quenching agent every 15 min.

Table S2. Temporal control over photo-ATRP of $OEOMA_{500}$.[a]

Entry	Time (min)	Light	Conv. (%)
1	0	OFF	0
2	0-15	ON	15
3	15-30	OFF	18
4	30-45	ON	65
5	45-60	OFF	65
6	60-75	ON	82
7	75-90	OFF	83
8	90-105	ON	89
9	105-120	OFF	91

[a]Reaction conditions: $[OEOMA_{500}]/[HO\text{-}EBiB]/[MB^+]/[CuBr_2]/[TPMA]$ = 200/1/0.025/0.3/0.9, $[OEOMA_{500}]$ = 300 mM, in 1X PBS with DMSO (10% *v*/*v*), red LEDs (630 nm, 25 mW cm^{-2}) were turned on/off periodically for every 15 min in a 96-well plate.

Mechanistic study (Figure 4)

Cary 100 UV-Vis two-beam spectrometer was used to record UV-Vis absorption spectra in the range from 800 to 200 nm with 1 nm step, using quartz cell. The fluorescence lifetimes were measured on a fluorescence lifetime spectrometer (FluoTime300 from PicoQuant) with a detection system based on time-correlated single-photon counting (TCSPC). The emission decay lifetimes were acquired using a 640 nm diode laser as the excitation source. In addition, an instrument response function (IRF, prompt) was obtained using Ludox solution (colloidal silica).

Singlet oxygen emission were carried out on a FluoTime 300 fluorescence spectrophotometer with a NIR PMT detector H10330–45 (Hamamatsu) equipped with a 1000 nm long-pass filter. The samples were excited at 660 nm using a high repetition rate 40 MHz picosecond laser diode (LDH-660 nm, PicoQuant). Data acquisition was performed using a computer-mounted PCI-board multichannel scaler (NanoHarp 250, PicoQuant). The MB^+ absorbance was adjusted to be 0.1 at the excitation wavelength. The time-resolved measurements (decay traces at λ = 1270 nm) were collected using a so-called “burst mode”, where the sample is first excited by multiple laser pulses to build up the population of singlet oxygen and then left to decay in the 100 μs time window. The experiment was performed in 1X PBS with DMSO (10% *v*/*v*). Quartz cells with an optical length of 10 mm were used for all measurements.

The setup for the nanosecond laser flash photolysis (LFP) experiments and the data acquisition system have been described in detail previously.[4] For LFP experiments, a Nd:YAG laser (532 nm, 2 mJ, 7–9 ns) was used for excitation. Transient decays were recorded at individual wavelengths by the step-scan method with a step distance of 10 nm in the range of 380 to 800 nm as the mean of 10 probe pulses. The experiments were performed in rectangular quartz cells (10 mm × 10 mm).

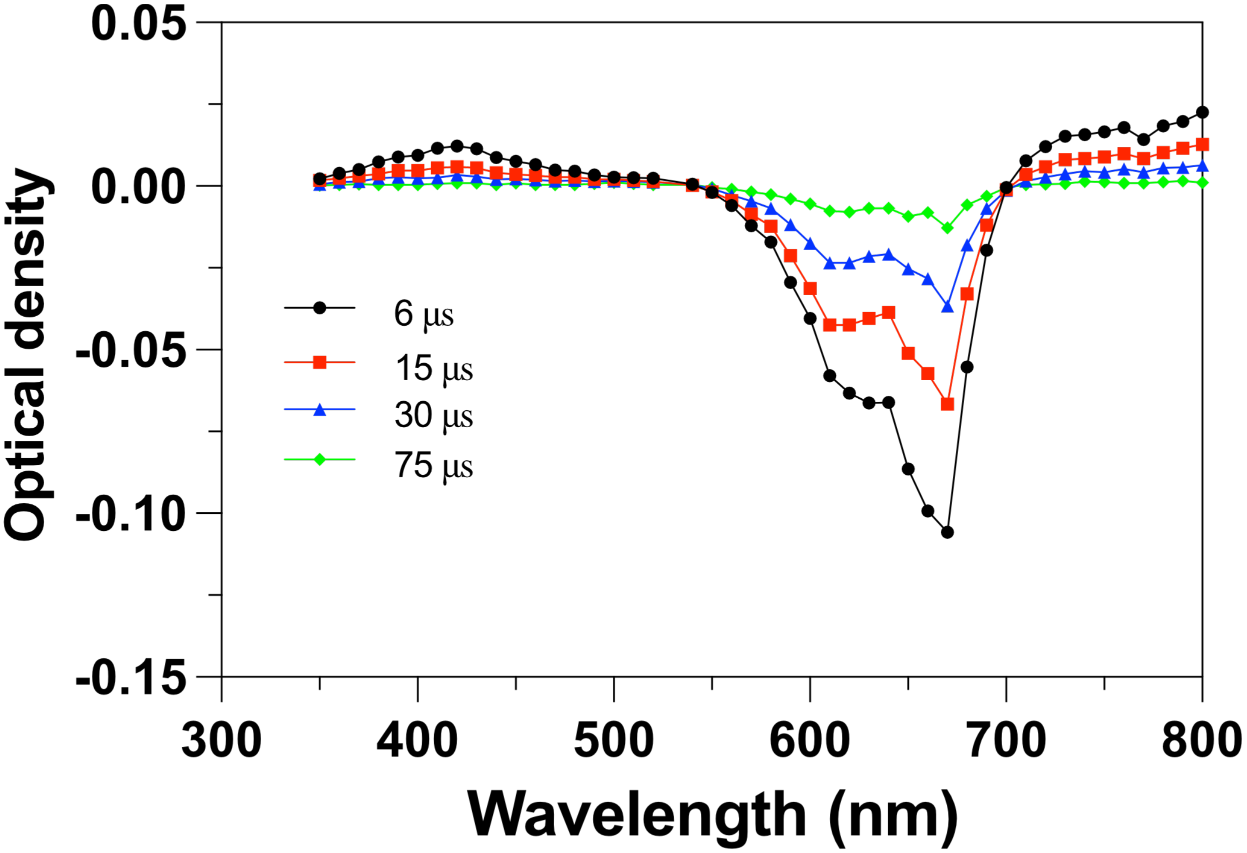

Figure S6. Transient UV-vis absorption spectra obtained during laser flash photolysis (with excitation at 532 nm) of deoxygenated 1X PBS with DMSO (10% *v*/*v*) of MB^+ (37.5 M); time delay after laser flash 6 μs to 70 μs.

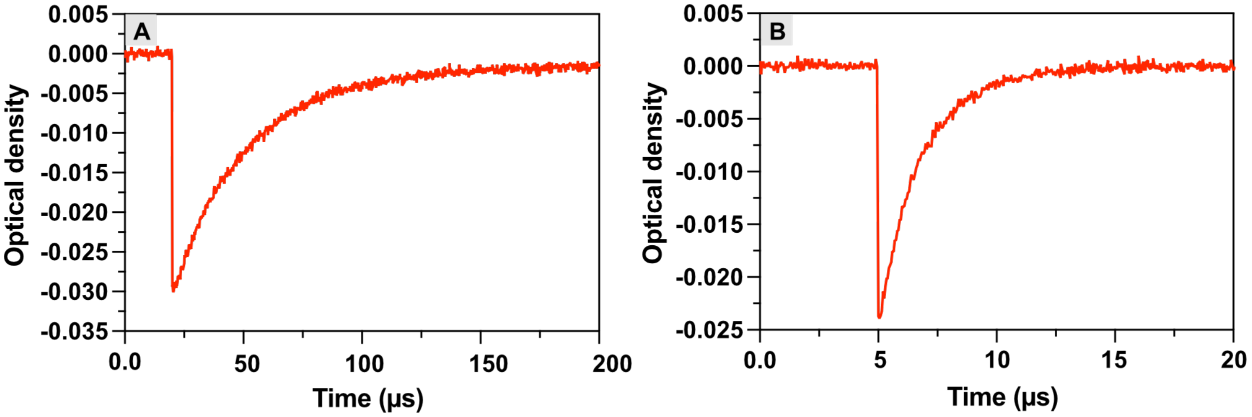

Figure S7. Decay profiles monitored at 640 nm obtained during laser flash photolysis (with excitation at 532 nm) of deoxygenated 1X PBS with DMSO (10% *v*/*v*) of MB^+ (37.5 μM) with (A) or without (B) argon purge.

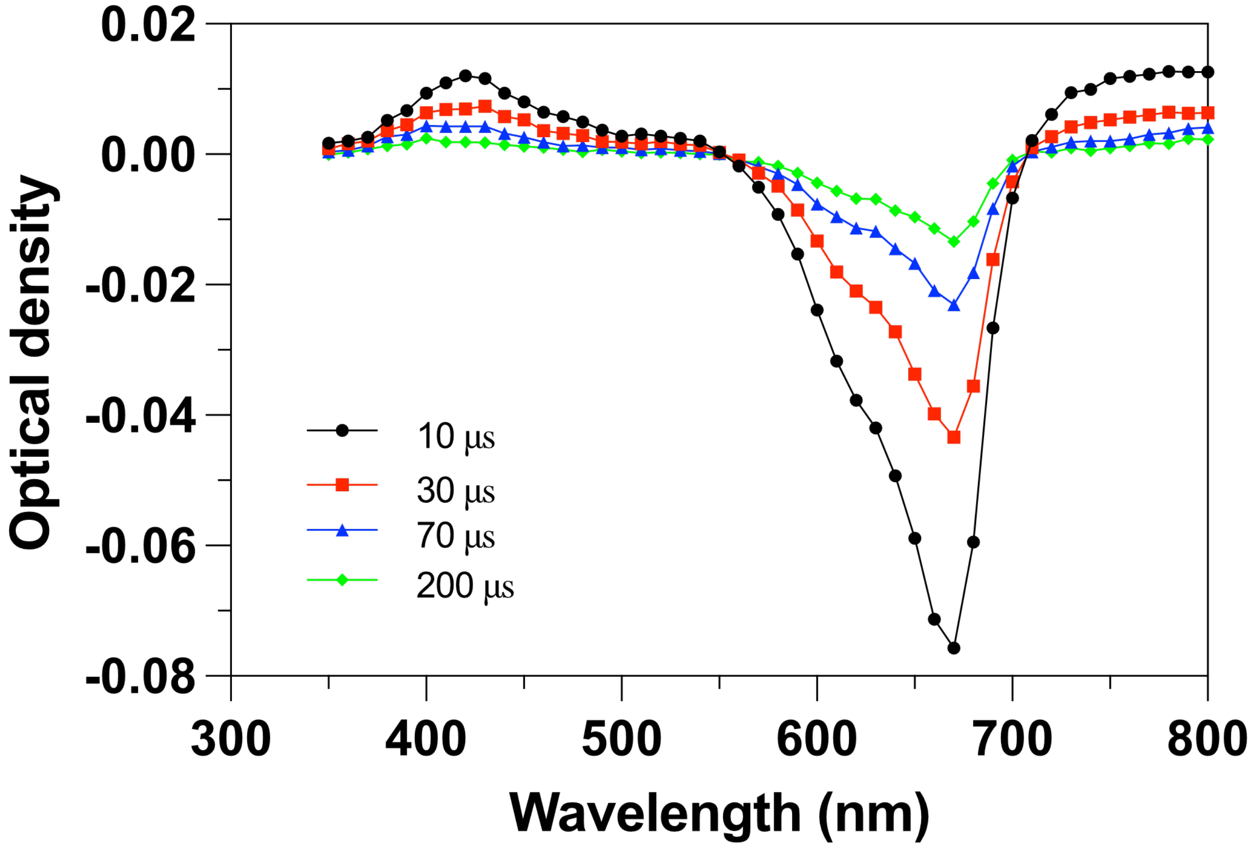

Figure S8. Transient UV-vis absorption spectra obtained during laser flash photolysis (with excitation at 532 nm) of deoxygenated 1X PBS with DMSO (10% *v*/*v*) of MB^+ (37.5 M) in the presence of TPMA; time delay after laser flash 10 μs to 200 μs.

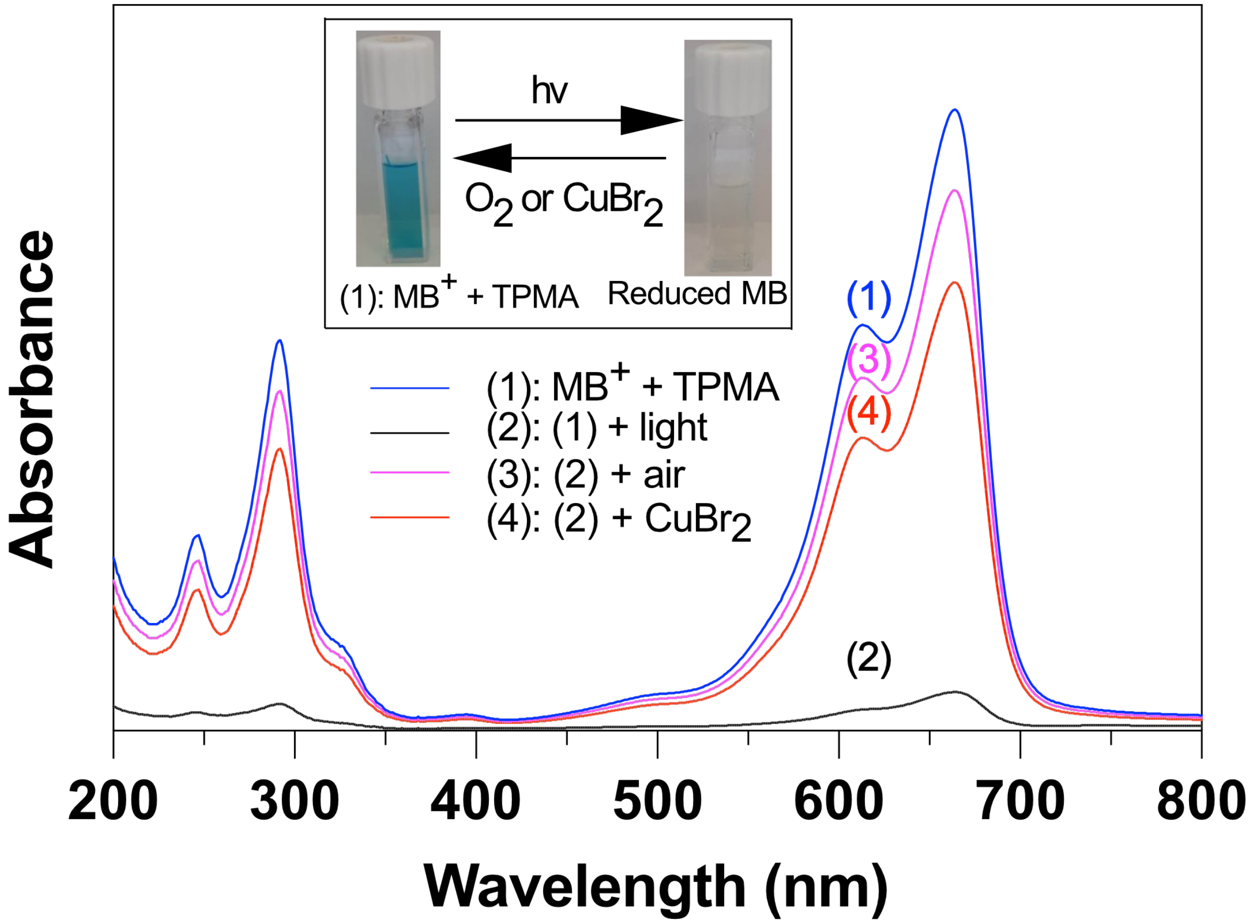

Figure S9. UV-vis absorption of MB^+ (23 μM) in the presence of TPMA (11 mM) before irradiation (blue), after steady-state irradiation with 640 nm LED (black), after purging with air (pink) or addition of air-free $CuBr_2$ (red).

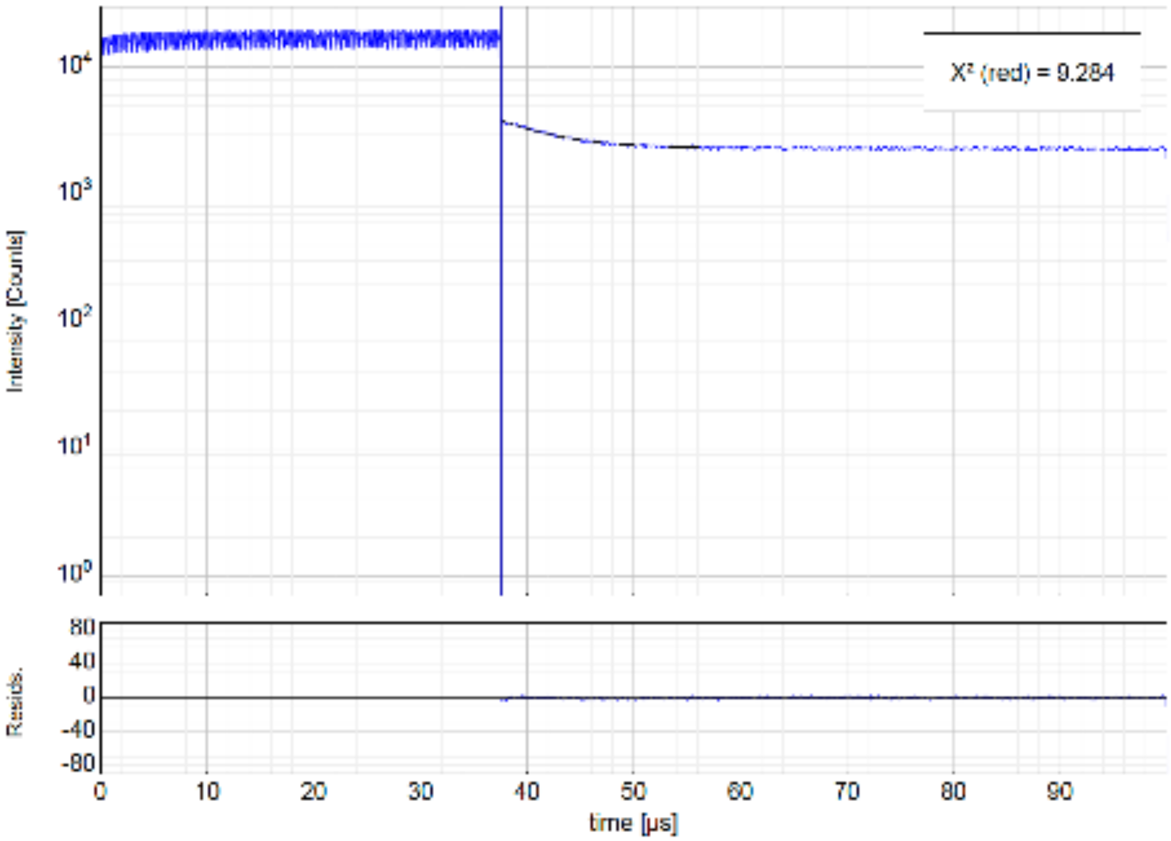

$$I(t) = \sum_{i=1}^{n} A_i e^{-\frac{t}{\tau_i}}$$

Parameter	Value	Conf. $_{Lower}$	Conf. $_{Upper}$	Conf. $_{Estimation}$
A_1 [Cnts]	1539	-128	+128	Fitting
τ_1 [µs]	5.683	-0.613	+0.613	Fitting
Bkgr. $_{Dec}$ [Cnts]	2281.6	-23.5	+23.5	Fitting

Figure S10. Decay curves and the fit of the singlet oxygen generated by free MB^+ in 1X PBS with DMSO (10% *v*/*v*) (λ_{exc}= 660 nm, λ_{em}= 1270 nm, collection time: 45 min).

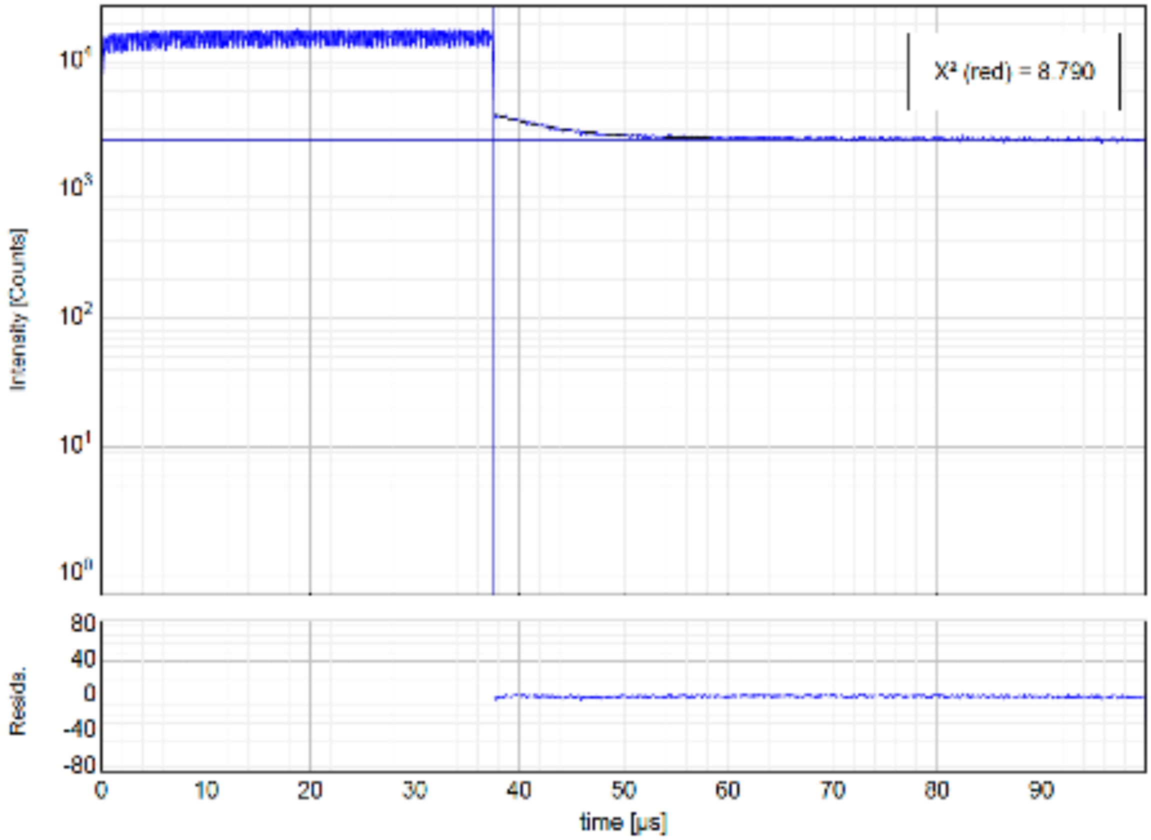

$$I(t) = \sum_{i=1}^{n} A_i e^{-\frac{t}{\tau_i}}$$

Parameter	Value	Conf. $_{Lower}$	Conf. $_{Upper}$	Conf. $_{Estimation}$
A_1 [Cnts]	1433	-121	+121	Fitting
τ_1 [µs]	6.349	-0.698	+0.698	Fitting
Bkgr. $_{Dec}$ [Cnts]	2489.9	-23.9	+23.9	Fitting

Figure S11. Decay curves and the fit of the singlet oxygen generated by MB+ in 1X PBS with DMSO (10% *v*/*v*) in the presence of TPMA (11 mM) (λ_{exc}= 660 nm, λ_{em}= 1270 nm, collection time: 45 min).

Calculation of Gibbs free energy for the photoinduced electron transfer

To determine the thermodynamic feasibility of two different mechanisms, the Gibbs free energy for the photoinduced electron transfer process was calculated for both the reductive and oxidative quenching cycles. Based on equation (1)

$$\Delta G_{et}\ (eV) = -[E_{1/2}(A/A^{\bullet -}) - E_{1/2}(D^{\bullet +}/D)] - E_{PC^*} + \Delta E \quad (1)$$

where $E_{1/2}(A/A^{\bullet -})$ is the reduction potential of an electron acceptor (A), $E_{1/2}(D^{\bullet +}/D)$ is the oxidation potential of a sacrificial electron donor (D), E_{PC^*} is the energy of the singlet or triplet excited state of the photocatalyst. In polar solvents, ΔE = < 0.1 eV, and is often neglected in photophysical estimations. Excited energy for $^3E_{MB^*}$ = 1.50 eV.[5]

For the oxidative quenching cycle:

$$\Delta G_{et}\ (V) = -[E_{1/2}(Cu^{II}/Cu^{I}) - E_{1/2}(MB^{\bullet 2+}/MB^{+})] - {}^3E_{MB^*} = -(-0.23-(-0.68))-1.50 = -1.95\ V \quad (2)$$

$$\Delta G\ (V) = -[\ E_{1/2}(MB^{\bullet 2+}/MB^{+})] - E_{1/2}(L^{\bullet +}/L)\ ] = -(1.13-0.96) = -\ 0.17\ eV \quad (3)$$

$$\Delta G_{total} = \Delta G_{et}\ (V) + \Delta G\ (V) = (-1.95) + (-\ 0.17) = -2.12\ eV \quad (4)$$

The electron transfer from MB^{+*} to $[Br-Cu^{II}/TPMA]^{+}$ gives a free energy value of ΔG_{et} = -1.95 V. The subsequent reduction of $MB^{\bullet 2+}$ by TPMA is also thermodynamically favored (ΔG = −0.17 eV) according to equation 3, without the need for additives. The total energy change for the oxidative quenching cycle is -2.12 eV.

For the reductive quenching cycle:

$$\Delta G_{et}\ (V) = -[E_{1/2}(MB^{+}/MB^{\bullet}) - E_{1/2}(L^{\bullet +}/L)] - {}^3E_{MB^*} = -(1.6-0.96)\ -1.5 = -2.14\ eV \quad (5)$$

$$\Delta G\ (V) = -[\ E_{1/2}(Cu^{II}/Cu^{I}) - E_{1/2}(MB^{\bullet}/MB^{+})] = -(-0.23-(-0.30)) = -0.07\ eV \quad (6)$$

$$\Delta G_{total} = \Delta G_{et}\ (V) + \Delta G\ (V) = (-2.14) + (-\ 0.07) = -2.21\ eV \quad (7)$$

The redox potential of TPMA is $E_{1/2}(R_3N^{\bullet +}/R_3N)$ = +0.96 V vs SCE based on the onset of its oxidation peak in MeCN, the electron transfer from TPMA to MB^{+*} gives a free energy value of ΔG_{et} = -2.14 eV. The subsequent reduction of $[Br-Cu^{II}/TPMA]^{+}$ by $MB^{\bullet}$ is also thermodynamically favored (ΔG = −0.07 eV) according to equation 5. The total energy change for the oxidative quenching cycle is -2.21 eV.

Based on the calculation, the energy change ΔG_{total} = -2.21 eV for the reductive quenching, -2.12 eV for the oxidative quenching, which shows good agreement as the energy change should be the same for the same reaction and independent of the reaction pathways.

Polymerization under different light wavelengths (Figure 5A)

ATRP "cocktail" solution (5 mL) was prepared according to the general procedure for MB^+/Cu-catalyzed photo-ATRP at concentrations of $OEOMA_{500}$ (300 mM), MB^+ (37.5 μM), $CuBr_2$ (0.45 mM), TPMA (1.35 mM), HO-EBiB (1.5 mM), DMSO (10% *v*/*v*), and 1X PBS. The solution was first transferred to a multichannel pipette reservoir (20 mL), and then to each well in a 96-well plate using a multichannel pipette. The ATRP "cocktail" solutions in a 96-well plate were irradiated under a different light (UV light: 395 nm, 30 mW cm^{-2}, blue light: 445 nm, 45 mW cm^{-2}, green light: 527 nm, 20 mW cm^{-2}, red light: 630 nm, 25 mW cm^{-2}, NIR light: 730 nm, 55 mW cm^{-2}, and sunlight) for 60 min. At different time intervals (0, 5, 10, 15, 20, 30, 40, 50, and 60 min), SnatchCat solution (50 μl, 1 mg/mL in H_2O) was added to the corresponding wells to stop the polymerizations. At the end of the polymerizations, all samples were withdrawn and analyzed by ^{1}H NMR and SEC techniques (DMF).

Polymerization at varying scale

Table S3. MB^+/Cu-catalyzed photo-ATRP in scale from 50 μL to 4.5 mL.[a]

Entry	Volume (μL)	Time (min)	Conv. (%)	$M_{n,th}$	$M_{n,app}$	$M_{n,MALS}$	*Đ*
1[a]	50	30	6	6 000	-	-	-
2[a]	100	30	54	54 000	52 300	88 800	1.19
3[a]	150	30	68	68 000	53 500	92 100	1.23
4[a]	200	30	73	73 000	46 700	93 400	1.21
5[a]	250	30	71	71 000	51 900	70 500	1.17
6[b]	50	30	31	31 000	30 600	31 000	1.19
7[c]	1800	60	56	56 000	42 400	49 000	1.19
8[c]	4500	60	47	47 000	36 500	53 000	1.18

[a]Reaction conditions: $[OEOMA_{500}]/[HO\text{-}EBiB]/[MB^+]/[CuBr_2]/[TPMA]$ = 200/1/0.025/0.3/ 0.9, $[OEOMA_{500}]$ = 300 mM Photo-ATRP in a [a]96-well plate, [a]irradiated under red LEDs (630 nm, 25 mW/cm^2) in a [b]pipette tip (200 uL), [c]1.8 ml and 4.5 mL solution in a 1-dram vial irradiated under red LEDs (630 nm, 2.5 mW cm^{-2}) in Evoluchem photoreactor. Molecular weight ($M_{n,app}$) and dispersity (*Đ*) were determined by SEC analysis (DMF as

eluent) calibrated to poly(methyl methacrylate) standards. Absolute molecular weight ($M_{n,MALS}$) was determined by SEC analysis (DMF as eluent) with a multi-angle light scattering (MALS) detector.

Polymerization at different light intensity

Table S4. MB+/Cu-catalyzed photo-ATRP at different light intensity.[a]

Entry	Light intensity (mW cm^{-2})	Conv. (%)	$M_{n,th}$	$M_{n,app}$	$M_{n,MALS}$	$Đ$
1	25	71	71 000	51 900	70 500	1.21
2	55	81	81 000	58 200	11 200	1.32
3	75	90	90 000	62 200	10 900	1.36
4	100	90	90 000	73 900	97 600	1.42
5	125	91	91 000	62 800	10 800	1.41

[a]Reaction conditions: [$OEOMA_{500}$]/[HO-EBiB]/[MB^+]/[$CuBr_2$]/[TPMA] = 200/1/0.025/0.3/ 0.9, [$OEOMA_{500}$] = 300 mM, in 1X PBS with DMSO (10% *v*/*v*), irradiated for 30 min under red LEDs (630 nm, X mW cm^{-2}) in a 96-well plate. Monomer conversion was determined by using ^{1}H NMR spectroscopy. Molecular weight and dispersity ($Đ$) were determined by GPC analysis (DMF as eluent) calibrated to poly(methyl methacrylate) standards. Absolute molecular weight ($M_{n,MALS}$) was determined by SEC analysis (DMF as eluent) with a multi-angle light scattering (MALS) detector.

Photobleaching of common dyes under different light (Figure 5B and C)

Stock solutions (0.1 mM) of DAPI and Cy3-alkyne were prepared by dissolving them in 1X PBS. 250 μL of the dye solution was transferred to a 96-well plate followed by irradiation with UV (395 nm, 30 mW cm^{-2}), green (527 nm, 20 mW cm^{-2}), and red (630 nm, 25 mW cm^{-2}) light. After 30 min of irradiation, the dye solutions were diluted with deionized water to the final concentration of 4 μM. 250 μL of the diluted solutions were transferred to a 96-well plate. The fluorescence intensity of DAPI (ex: 358 nm, em: 458 nm) and Cy3-alkyne (ex: 555 nm, em: 570 nm) were recorded by using a microplate reader (*The Infinite® M1000, Tecan*). The average fluorescence intensity and the standard deviation were calculated from triplicate experiments.

Polymerization in DMSO

First, the stock solutions of MA (8.0 M in DMSO), MB^+ (6.88 mM in DMSO), EBiB (200 mM in DMSO), $CuBr_2$ (56.24 mM in DMSO), and Me_6TREN (336.33 mM in DMSO) were prepared. As a typical example of the synthesis of pMA (entry 2, Table S5), the ATRP "cocktail" (250 µL) was prepared as follows. MA stock (171.88 µL), MB^+ stock (5 µL), $CuBr_2$ stock (2.44 µL), Me_6TREN stock (2.45 µL), EBiB stock (34.37 µL), and DMSO (33.86 µL) were mixed. The final concentrations were MA (5.5 M), MB^+ (137.5 µM), $CuBr_2$ (0.55 mM), Me_6TREN (3.3 mM), and EBiB (27.5 mM). The ATRP "cocktail" was then transferred to a glass insert. The polymerization mixture was irradiated under red LEDs (630 nm, 2.5 mW cm^{-2}) for 60 min. Samples were taken for ^1H NMR and SEC characterization (THF eluent).

Table S5. MB^+/Cu-catalyzed photo-ATRP in DMSO.[a]

Entry	$[MB^+]$ (equiv)	$[CuBr_2]$ (equiv)	Conv. (%)	$M_{n,th}$	$M_{n,app}$	$Đ$
1	0.005	0.05	28	5 011	7700	1.32
2	0.005	0.02	90	15 675	16 200	1.09
3	0.001	0.02	68	11 891	14 400	1.10
4	0.0005	0.02	1	-	-	-

[a]Reaction conditions: $[MA]/[EBiB]/[MB^+]/[CuBr_2]/[Me_6TREN]$ = 200/1/x/y/6y, [MA] = 5.5 M, irradiated for 60 min under red LEDs (630 nm, 2.5 mW cm^{-2}) in a glass jar. Molecular weight and dispersity ($Đ$) were determined by GPC analysis (THF as eluent) calibrated to poly(methyl methacrylate) standards.

Synthesis of DNA-polymer bioconjugate (Figure 6)

The stock solution of DNA-bearing ATRP initiator (T_{10}-Br, 10 mM in H_2O) was prepared based on our previous report.[2] $OEOMA_{500}$ stock (125 µL), MB^+ stock (5 µL), $CuBr_2$ stock (10 µL), TPMA stock (5 µL), T_{10}-Br stock (18.75 µL), DMSO (15 µL), H_2O (65 µL), and 10X PBS solution (25 µL) were mixed by vortex and transferred to a 96-well plate. The final concentrations were $OEOMA_{500}$ (300 mM), MB^+ (37.5 µM), $CuBr_2$ (0.45 mM), TPMA (1.35 mM), and T_{10}-Br (0.75 mM). The plate containing the ATRP "cocktail" solution was mounted on the Lumidox® Gen II 96-point LED array. The polymerization mixtures were

irradiated under red LEDs (630 nm, 25 mW cm^{-2}) for 30 min. At the end of the polymerization, the samples were withdrawn and analyzed by ^1H NMR and SEC technique (DMF eluent).

Biocompatibility of MB^+/Cu-catalyzed photo-ATRP in presence of cells (Figure 7)

Cytotoxicity was evaluated by live/dead cell assay according to the protocol from the manufacturer (*Thermo Fisher Scientific).* First, a live/dead assay stain was prepared by dissolving calcein AM (5 µL) and ethidium homodimer-1 (20 µL) in 1X PBS (10 mL). ATRP "cocktail" solution (5 mL) was prepared according to the general procedure for MB^+/Cu-catalyzed photo-ATRP at final concentrations of $OEOMA_{500}$ (300 mM), MB^+ (37.5 µM), $CuBr_2$ (0.45 mM), TPMA (1.35 mM), HO-EBiB (1.5 mM), DMSO (0.27% *v*/*v*), and PBS (1X). Aliquots of the NIH3T3 cell (fibroblast cell line, 100 µL) suspension containing 40 000 cells/mL were transferred to the wells of 96-well microplate and allowed to adhere overnight. After the cells were attached, the media was aspirated, and the cells were washed with 1X PBS.

In two identical 96-well plates containing cells, the ATRP "cocktail" solution (250 µL) or 1X PBS (as the control group) was added to the cells in 96-well plates. The polymerization mixtures were irradiated under red LEDs (630 nm, 25 mW cm^{-2}) for 5 min. Noting that the polymerization time was set to 5 min to minimize light-induced cell-death based on previous work.[6] Another plate was kept in the dark by being covered with aluminium foil. After 5 min, cells were washed with 1X PBS and stained with the prepared dye solution for 30 min. After 30 min of incubation, the fluorescence intensity from the calcien AM and ethidium homodimer-1 dye was measured using the TECAN spectrophotometer reader. By normalizing the fluorescence intensity to the control group PBS in the dark, the cytotoxicity of the cell incubated with ATRP "cocktail" with or without light irradiation and PBS as the control group with light irradiation was calculated.

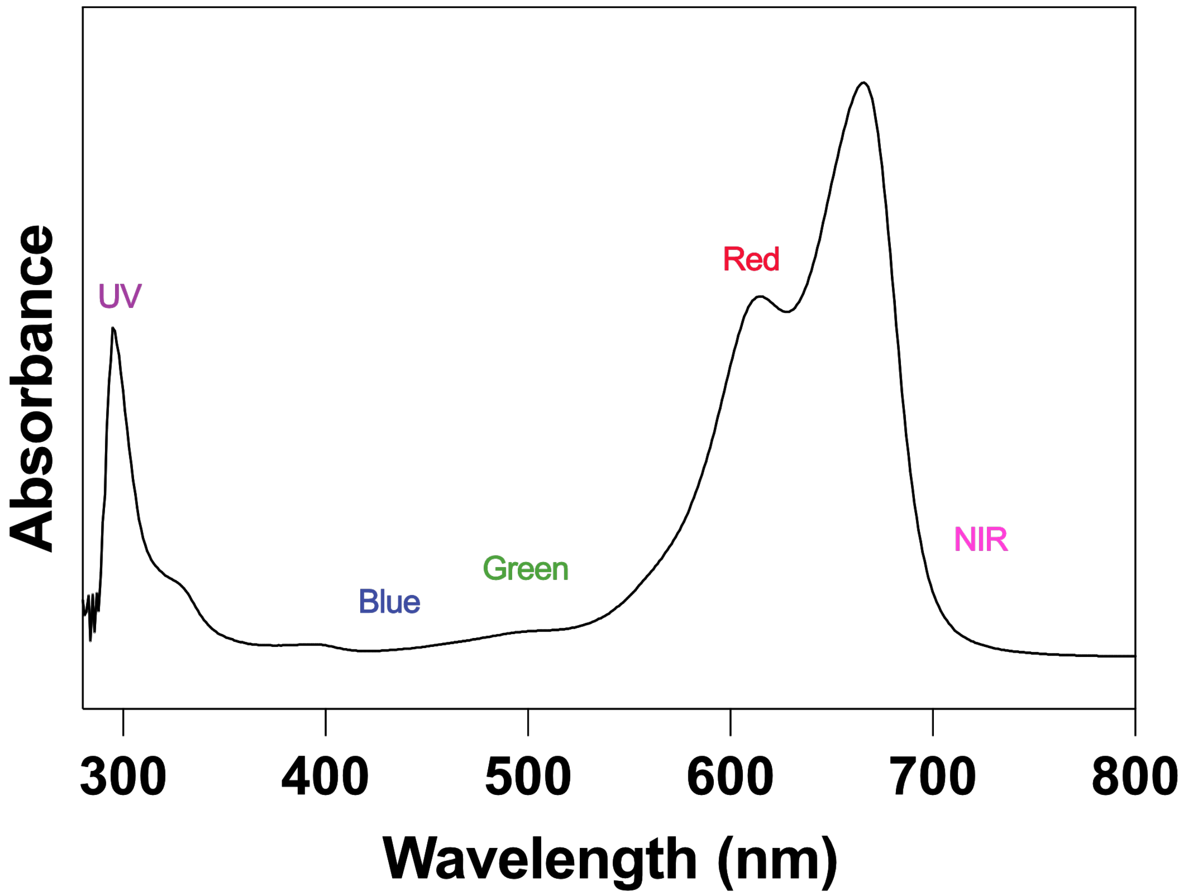

Figure S12. UV−vis spectrum of MB^+.

References

1. Murata, H.; Cummings, C. S.; Koepsel, R. R.; Russell, A. J., Rational Tailoring of Substrate and Inhibitor Affinity via ATRP Polymer-Based Protein Engineering. *Biomacromolecules* **2014,** *15* (7), 2817-2823.
2. Averick, S. E.; Dey, S. K.; Grahacharya, D.; Matyjaszewski, K.; Das, S. R., Solid-Phase Incorporation of an ATRP Initiator for Polymer–DNA Biohybrids. *Angew. Chem. Int. Ed.* **2014,** *53* (10), 2739-2744.
3. Szczepaniak, G.; Piątkowski, J.; Nogaś, W.; Lorandi, F.; Yerneni, S. S.; Fantin, M.; Ruszczyńska, A.; Enciso, A. E.; Bulska, E.; Grela, K.; Matyjaszewski, K., An isocyanide ligand for the rapid quenching and efficient removal of copper residues after Cu/TEMPO-catalyzed aerobic alcohol oxidation and atom transfer radical polymerization. *Chem. Sci.* **2020,** *11* (16), 4251-4262.
4. Pedzinski, T.; Markiewicz, A.; Marciniak, B., Photosensitized oxidation of methionine derivatives. Laser flash photolysis studies. *Res. Chem. Intermed.* **2009,** *35* (4), 497-506.
5. Romero, N. A.; Nicewicz, D. A., Organic Photoredox Catalysis. *Chem. Rev.* **2016,** *116* (17), 10075-10166.
6. Niu, J.; Lunn, D. J.; Pusuluri, A.; Yoo, J. I.; O'Malley, M. A.; Mitragotri, S.; Soh, H. T.; Hawker, C. J., Engineering live cell surfaces with functional polymers via cytocompatible controlled radical polymerization. *Nat. Chem.* **2017,** *9* (6), 537-545.
